# Comparison between 16S rRNA and shotgun sequencing in colorectal cancer, advanced colorectal lesions, and healthy human gut microbiota

**DOI:** 10.1186/s12864-024-10621-7

**Published:** 2024-07-29

**Authors:** David Bars-Cortina, Elies Ramon, Blanca Rius-Sansalvador, Elisabet Guinó, Ainhoa Garcia-Serrano, Núria Mach, Olfat Khannous-Lleiffe, Ester Saus, Toni Gabaldón, Gemma Ibáñez-Sanz, Lorena Rodríguez-Alonso, Alfredo Mata, Ana García-Rodríguez, Mireia Obón-Santacana, Victor Moreno

**Affiliations:** 1 Unit of Biomarkers and Susceptibility (UBS), Oncology Data Analytics Program (ODAP), Catalan Institute of Oncology (ICO), L’Hospitalet del Llobregat, Barcelona, 08908 Spain; 2 ONCOBELL Program, Bellvitge Biomedical Research Institute (IDIBELL), L’Hospitalet de Llobregat, Barcelona, 08908 Spain; 3 Doctoral Programme in Biomedicine, University of Barcelona (UB), Barcelona, 08907 Spain; 4 Consortium for Biomedical Research in Epidemiology and Public Health (CIBERESP), Madrid, 28029 Spain; 5 Department of Clinical Science, Intervention and Technology, Karolinska Institutet, Stockholm, 14186 Sweden; 6 IHAP, Université de Toulouse, INRAE, ENVT, Toulouse, France; 7 Barcelona Supercomputing Centre (BSC-CNS), Barcelona, 08034 Spain; 8 Institute for Research in Biomedicine (IRB Barcelona), The Barcelona Institute of Science and Technology, Barcelona, 08028 Spain; 9 Catalan Institution for Research and Advanced Studies (ICREA), Barcelona, 08010 Spain; 10 Centro de Investigación Biomédica en Red de Enfermedades Infecciosas (CIBERINFEC), Barcelona, 08028 Spain; 11 Gastroenterology Department, Bellvitge University Hospital, L’Hospitalet de Llobregat, Barcelona, 08907 Spain; 12 Digestive System Service, Moisés Broggi Hospital, Sant Joan Despí, 08970 Spain; 13 Endoscopy Unit, Digestive System Service, Viladecans Hospital-IDIBELL, Viladecans, 08840 Spain; 14 Department of Clinical Sciences, Faculty of Medicine and Health Sciences, Universitat de Barcelona Institute of Complex Systems (UBICS), University of Barcelona (UB), L’Hospitalet de Llobregat, Barcelona, 08908 Spain

**Keywords:** Microbiome, 16S, Shotgun, Colorectal cancer, Comparison

## Abstract

**Background:**

Gut dysbiosis has been associated with colorectal cancer (CRC), the third most prevalent cancer in the world. This study compares microbiota taxonomic and abundance results obtained by 16S rRNA gene sequencing (16S) and whole shotgun metagenomic sequencing to investigate their reliability for bacteria profiling. The experimental design included 156 human stool samples from healthy controls, advanced (high-risk) colorectal lesion patients (HRL), and CRC cases, with each sample sequenced using both 16S and shotgun methods. We thoroughly compared both sequencing technologies at the species, genus, and family annotation levels, the abundance differences in these taxa, sparsity, alpha and beta diversities, ability to train prediction models, and the similarity of the microbial signature derived from these models.

**Results:**

As expected, the results showed that 16S detects only part of the gut microbiota community revealed by shotgun, although some genera were only profiled by 16S. The 16S abundance data was sparser and exhibited lower alpha diversity. In lower taxonomic ranks, shotgun and 16S highly differed, partially due to a disagreement in reference databases. When considering only shared taxa, the abundance was positively correlated between the two strategies. We also found a moderate correlation between the shotgun and 16S alpha-diversity measures, as well as their PCoAs. Regarding the machine learning models, only some of the shotgun models showed some degree of predictive power in an independent test set, but we could not demonstrate a clear superiority of one technology over the other. Microbial signatures from both sequencing techniques revealed taxa previously associated with CRC development, e.g., *Parvimonas micra*.

**Conclusions:**

Shotgun and 16S sequencing provide two different lenses to examine microbial communities. While we have demonstrated that they can unravel common patterns (including microbial signatures), shotgun often gives a more detailed snapshot than 16S, both in depth and breadth. Instead, 16S will tend to show only part of the picture, giving greater weight to dominant bacteria in a sample. Therefore, we recommend choosing one or another sequencing technique before launching a study. Specifically, shotgun sequencing is preferred for stool microbiome samples and in-depth analyses, while 16S is more suitable for tissue samples and studies with targeted aims.

**Supplementary Information:**

The online version contains supplementary material available at 10.1186/s12864-024-10621-7.

## Background

Colorectal cancer (CRC) is the second deadliest cancer and the third most common malignancy worldwide [1]. It is estimated that 80% of CRC cases are not heritable [2] and take place following the adenoma-carcinoma sequence (ACS) that involves the progressive accumulation of mutations in a period of 10–15 years on average [3]. Apart from being older than 50 years, there are modifiable (e.g., lifestyle and dietary habits) and non-modifiable (e.g., type 2 diabetes) risk factors for CRC [4]. Moreover, the gut microbiota is one active player that influences the CRC development [5, 6], and their characterization is continuously improving in pursuit of candidate screening biomarkers [7, 8]. The dynamics of the microbiota studied throughout ACS and the prediction of different stages of CRC development allow the identification of certain microbiological taxa of interest: the ‘microbial signature’. Bacteria widely associated to CRC include several *Fusobacterium* species [7, 9–13], *Parvimonas micra* [7, 10, 11, 13, 14], *Porphyromonas asaccharolytica* [9–11], *Bacteroides fragilis* [10, 12, 13].

Traditionally, microbial communities have been characterized using culture-dependent methods, yet over 70% of human gut microbiome species remain uncultured [15]. Advances in high-throughput sequencing, such as 16S rRNA gene sequencing and shotgun whole genome sequencing, have broadened our understanding beyond what is possible with laboratory isolation alone [16]. The 16S rRNA gene sequencing, which targets nine hypervariable regions, remains the most widely used method for profiling microbial communities [17]. However, reliance on specific regions (usually V3-V4) for PCR primer design can introduce biases, as no single region can adequately distinguish all species [17, 18]. Additionally, variability in the number of 16S rRNA gene copies and within-genome differences can affect the accuracy of this method [19]. While 16S rRNA sequencing offers a cost-effective and computationally efficient approach for microbial identification, shotgun sequencing is becoming increasingly attractive. Traditionally, shotgun sequencing required more resources and incurred higher costs. However, recent advancements have narrowed the cost gap, making it a more viable option for many labs. Despite its established role, 16S sequencing may face growing competition from shotgun sequencing as affordability continues to improve.

In contrast to the targeted approach of 16S, shotgun sequences all genomes and genomic regions present in a given sample. Covering genomic regions outside the small 16S rRNA gene means that specific strain-level discrimination is achievable. Furthermore, shotgun can identify viruses, fungi, protozoa, bacteria, archaea, and other microorganisms. Currently, the greatest disadvantages of this sequencing technique are its higher cost, the noisy signal due to host contamination, and the need for a more intensive and complex bioinformatics analysis. Shotgun sequencing analysis is strongly dependent on the reference genome database [20], which may induce biases when many reads map to unknown taxa, especially when working with samples from complex or scarcely studied environments such as floodplain [21, 22] However, in studies focusing on the human gut microbiota this problem can minimized with the use of specialized databases.

Both techniques are widely employed in microbiome research and have their advantages and drawbacks, as depicted previously. However, researchers using these distinct methods often encounter difficulties reconciling their results. In addition to the differing resolution and potential sequencing biases discussed above, a key problem lies in the existence of distinct reference databases for each methodology (e.g., SILVA, Greengenes and RDP for 16S, and NCBI refseq, GTDB, UHGG, for shotgun). These databases differ significantly in size, update periodicity, content, and how they are curated [23]. Due to all these challenges, direct method comparisons between 16S and shotgun for human samples are limited [18]. Most of the available literature on this topic [16, 24–27] focuses on a “classical” view comparing taxonomic agreement, alpha-diversity values and beta-diversity projections generated by both techniques. Other studies [9, 28, 29] contrast the performance and microbial signature given by machine learning (ML) prediction models trained in shotgun and 16S data. Typically, comparisons are performed at the genus level, at least for 16S.

In this study, we apply several statistical models and methods developed for prokaryote profiling using 156 human stool samples. Our main objective is to perform a thorough comparison of 16S and shotgun sequencing technologies that includes both a classical ecological analyses like alpha and beta diversity measures or Permutational Analysis of Variance (PERMANOVA), and diverse ML approaches aiming to predict advanced (high-risk) colorectal lesions (HRL) and CRC.

## Methods

### Sample collection

The research cohort was recruited among individuals who participated in the COLSCREEN study [13]. Participants were men and women aged 50–69, invited to the ongoing population-based CRC screening program conducted during 2016–2020 by the Catalan Institute of Oncology in L’Hospitalet del Llobregat, Barcelona, Spain. Some clinically detected CRC cases were also included to enrich this group, because cancer incidence is a rare event in screening programs. One week prior to the colonoscopy preparation, participants were instructed to store a fecal sample at home at a temperature of -20 °C. On the day of the colonoscopy, participants delivered the stored sample which was preserved at -80 °C. The samples from all participants, including those from clinically detected CRC cases, were collected using the same standardized protocol, stored under identical conditions, and sequenced together in the same batch. Individuals were categorized based on the criteria commonly employed in CRC screening programs for risk assessment [30]. For this study, a subset of 156 cases belonging to the following three categories was selected: no-lesions/controls (*n* = 51), HRL (*n* = 54), and CRC cases (*n* = 51). Each stool sample was processed and sequenced with both shotgun and 16S techniques. The research protocol was approved by the ethics committee of Bellvitge University Hospital under the reference PR084/16.

### DNA extraction and sequencing

The fecal DNA was extracted using the NucleoSpin Soil Kit (Macherey-Nagel, Duren, Germany) following the manufacturer’s instructions for shotgun analysis [13] and through Dneasy PowerLyzer Powersoil kit (Qiagen, ref. QIA12855) for 16S [31]. The details for shotgun whole genome metagenomic sequencing and 16S rRNA amplicon sequencing are detailed in Obón-Santacana et al., 2022 [13] and Khannous-Lleiffe et al., 2022 [32] respectively.

### Bioinformatics analysis

For 16S, we applied our in-house bioinformatic pipeline to increase the proportion of amplicon sequence variants classified to the species level, in contrast to the conventional strategy [23]. The 16S data was preprocessed as previously described [32]. In brief, the 16S rRNA gene hypervariable V3-V4 region amplicon data were processed and analyzed using DADA2 v1.22.0 [33]. Low-quality reads were filtered and trimmed based on the observed quality profiles using the *filterAndTrim* function, truncating forward and reverse reads below 290 and 230, respectively, and considering a value of 2 as the maximum expected error. Furthermore, the first 10 nucleotides of each read were removed. We combined identical sequencing reads into unique sequences, made a sample inference from the matrix of estimated learning errors, and merged paired reads. For the sample inference step, the argument of the pool was defined as True. Chimeras and contaminants are often rare but spread across samples, making them more effectively identified when the samples are pooled (pool = T). Chimeric sequences were removed by using the *removeBimeraDenovo* function and taxonomy was assigned utilizing the SILVA 16S rRNA database (v138.1). Subsequently, to increase the percentage of Amplicon Sequence Variants (ASV) classified up to species level, from the ASV sequences obtained through DADA2, an additional taxonomic classification was performed using custom BLASTN database constructed from the SILVA database version stated above and performing an extra taxonomical classification based on *k*-mers (Kraken2 and Bracken2) using the NCBI RefSeq Targeted Loci Project database. Only 2.2% of ASVs could not be found in the reference database and were excluded from further analyses. For approximately one-fifth of the lineages we found more than one candidate species. These ASVs are named after all potential candidates, separated by “/” and alphabetically arranged (e.g., *Blautia obeum/wexlerae*).

Concerning shotgun, raw data in fastq format were processed to filter out human sequence reads (using human genome GRCh38) through Bowtie2 (v2.3.4) with options –very-sensitive-local and -k 1. A fastq file was then generated from reads that did not align (carrying SAM flag 12) using Samtools (v1.8). Then, once human sequences were removed, quality procedures and filters were applied. In detail, sequences were deduplicated using clumpify from the BBTools suite (v38.26), followed by a quality trimming (PHRED > 20) on both ends, sequencing adapters removal, and exclusion of read pairs where one read had a length lower than 75 bases using BBDuk (BBTools suite). In addition, with fastqcr v0.1.2 R package, FASTQC (v0.11.7), and Multi-QC (v1.12), a detailed analysis and snapshot of the quality control process was tackled. After the cleansing process, the reads were classified using Kraken2 (v2.1.2), with a filtering threshold of 0.1, followed by Bayesian re-assignment at the species level using Bracken2 (v2.2), with the read length parameter set at 150. The database used for the taxonomy assignment corresponded to prokaryotic data from the UHGG database v2.0.1 [34]. We selected this database that is specific for human gut microbiome to reduce false assignments that we experienced in previous attempts with the default NCBI RefSeq database that Kraken2 uses. We excluded fungi, viruses, and protozoa and focused on bacteria and archaea for the shotgun and 16S comparison. Only 11.8% (± 2.04%) of reads per sample could not be mapped to the UHGG database and were excluded from the subsequent analyses.

Finally, we ensured an adequate matching of taxonomical lineages between both techniques because the DADA2 pipeline for 16S used SILVA v138.1 + NCBI RefSeq Target Loci, while for shotgun we had selected UHGG v2.0.1. A thorough search allowed identifying discrepancies, mostly due to outdated taxa names, which were corrected and homogenized according to the NCBI taxonomy (16th March 2023). We used R packages (*myTAI* v0.9.3 and *taxonomizr* v0.10.2) for this task.

### Data pre-processing

The shotgun count matrix was normalized by genome length. Besides the original, species-level data, we also performed comparisons of the count matrices aggregated at genus and family taxonomic ranks. Some of the analyses (taxonomic overlap between shotgun and 16S, alpha diversity) were performed using the unfiltered abundance dataset, but the rest were performed after filtering all the species that were not present in at least 5% of the samples with 0.1% abundance or higher. To compare shotgun vs. 16S regarding sparsity, we computed the percentage of zeros by sample before and after filtering. In general, we favored a compositional treatment of the abundance matrices, as recommended for microbiome data [35]. Since metagenomic data are sparse, i.e., the taxa count matrix contains many zeros, zero-value replacement was performed using the square root Bayesian-Multiplicative method implemented in the R package *zCompositions* (v1.4.0–1). For the beta-diversity analysis, PERMANOVA, and ML models, we used the centered log ratio (clr)-transformation of this filtered and zero-replaced abundance matrix.

During this step, we noticed that, in 16S data, some candidate species appeared in more than one ASV (e.g., *Eubacterium callanderi/limosum*,* Eubacterium limosum/maltosivorans*). To avoid ambiguity in the microbial signature, we aggregated these taxa (and then applied the aforementioned data filtering, zero-replacement and clr-transformation) before training the 16S ML models.

### Statistical analysis

Statistical analyses were performed using R v4.2.2. The global overlap between shotgun and 16S within a given taxonomy rank (i.e. taxa detected by both methods) was visualized with Venn diagrams. We excluded from this comparison the unnamed fraction of taxa from both methods that was only identified by accession codes in the reference database (for instance, in shotgun, those species that start with MGYG). Then, we studied the correlation (Spearman) of the average abundance of taxa shared by shotgun and 16S. To this effect, we replaced the zero values and computed the closed geometric means (centre of compositional variable [36]) of the species, genus and families datasets separately for 16S and shotgun. For this analysis, we considered only taxa that were present in both sequencing methods. Then, we computed the geometric mean of the relative abundance of the taxa found by both sequencing method across samples and we ordered them in decreasing order to select the top 50 most prevalent taxa for shotgun and 16S. These were represented in heatmaps (*heatmap.2* function of v3.1.3 of package *gplots*). In addition to these analyses over “averaged” data, as the 156 stool samples were sequenced through both 16S and shotgun methods, we compared the taxonomic overlap and abundance profiles per sample. Moreover, taxon abundance agreement was assessed by the unweighted Cohen’s Kappa coefficient [37] after defining new binary variables for each taxon: 0 for those samples with less or equal counts than the median of that taxon and 1 to those with counts greater than the median. Furthermore, for all 156 samples, the top 15 most abundant species, genera and families detected by each of the two sequencing methods were represented in bar plots (*comp_barplot* function of v0.10.8 of *MicroViz* package). The order of the samples was the same in both sequencing methods, according to the Bray-Curtis dissimilarity distance [38] of 16S samples. To better identify the common shotgun-16S taxa, the same color legend is used in shotgun and 16 S barplot. We highlighted with “*” the species, genera and families with a prevalence > 90% (i.e. the core microbiota [39]).

Alpha diversity of the samples was computed from the raw data tables using two measures: Shannon (v2.6-4 package *vegan*) and Chao 1 Diversity Indexes (v0.4.0 package *fossil*). In order to allow a proper Shannon Index comparison [40], we adjusted the differences regarding the number of reads across the samples rarefying to the minimum number of reads in each method: the shotgun data at the value of 2,813,748 and 16S at 6,619. The Rarefaction Efficiency Indexes [41] were 0.94 and 0.92 respectively. As Chao 1 takes into account the unobserved species, it was calculated directly from raw abundance data [42]. Alpha diversity measures were represented with boxplots to facilitate the comparison of their distributions between the two sequencing methods. Additionally, scatterplots were used to visually illustrate the association between alpha diversity values obtained from shotgun and 16S sequencing for each sample. The strength of the association is measured by Spearman’s correlation coefficient [43] and represented by a regression line. Furthermore, the alpha diversity comparison between sequencing methods was statistically tested with the Wilcoxon Rank Sum Test.

We selected the Aitchison distance as beta-diversity measure due to its compatibility with compositional data analysis (Gloor et al., 2017), computed as the Euclidean distance over the clr-transformed data. Then, a principal coordinates analysis (PcoA) of this distance matrix was used to visualize the relationships between the 156 samples. Additionally, we conducted an Analysis of Similarities (ANOSIM) using the vegan v2.6-4 R library to quantify the similarities, and a PERMANOVA using the same library to test for differences (adjusted *p*-value < 0.05) among the three diagnostic groups (controls, HRL, and CRC). A post-hoc analysis of PERMANOVA was performed through the function *pairwise.perm.manova* (*RVAideMemoire* R package) and the multiple test correction of Benjamini and Hochberg was applied. Next, we computed the correlation between the 16S and shotguns’ PcoA projections with Procrustes *r* (library *vegan*) and Co-inertia RV coefficient (library *ade4* v1.7-22) and used a permutation test for significance. To visually represent the relation between the 6 PCoAs, we projected the matrix **K** of pairwise RV coefficients onto a principal component analysis (PCA). First, we computed the singular value decomposition of **K**, which equals to **K=**𝐔𝚲^2^𝐔^T^, as **K** is a squared, symmetric, and positive semidefinite matrix. Here, 𝚲^2^ is the diagonal matrix that contains the singular values of **K**, and **U** is an orthogonal matrix that contains the eigenvectors in the columns. Then, the PCA projections were obtained as 𝐔𝚲.

### Machine learning models

We used the clr-transformed abundance data to train ML algorithms predicting the diagnosis label: controls/HRL/CRC. We opted for linear SVM (*kernlab* package v0.9-31) and the nonlinear RF (*randomForest* package v4.7-1.1) methods. Our main objective was to compare if the shotgun and 16S data were able to fit similar classification models. To do so, we studied four items:

1. Training and test accuracy, measured as
  $$\:Accuracy\:=100\:\frac{correct\:predictions}{total\:predictions}\:$$
  when computing the training accuracy, “predictions” were the labels (controls/HRL/CRC) fitted by the model.

2. The shotgun-16S concordance with respect to the model fit and test prediction. This was measured with Cohen’s Kappa and with classification agreement, computed as
  $$Agreement = \:\frac{{number\,of\,shotgun\,predictions\,equal\,to\,16\,S\,predictions}}{{\:total\,predictions}}$$
  when comparing model fits, “predictions” correspond to the labels fitted in training. When working with SVM models we also computed the correlation between 16S and shotgun predictions. (Recall that every SVM model returns a numeric output that is binarized as: y > 0 → positive class, y < 0 → negative class)

3. (only in SVM) The matching of the shotgun-16S support vectors (SV), i.e. the subset of training samples that define the classification hyperplane. In addition to Cohen’s kappa, we computed the SV agreement following the previous formula (in this case, “predictions” corresponded to SV).
4. The similarity of the shotgun and 16S microbial signatures. To do so, we recovered the importance given by the models to each taxon and ranked the taxa from most to least important. The similarity was computed with the Kendall rank correlation coefficient between the shotgun and 16S rankings. Finally, we established the microbial signatures as the topmost important taxa of each model and computed the “agreement” as the percentage of taxa present in both shotgun and 16S signatures.

The training/test splitting was done via stratified random sampling. The test set for all models consisted of the same 30 individuals (*N* controls = 10, *N* HRL = 10, *N* CRC = 10), while the remaining 126 individuals (*N* controls = 41, N HRL = 44, N CRC = 41) were used for training. Hyperparameter optimization was done via 5 x cross-validation in the training set. For SVM, we optimized the cost using candidate values {0.1,1,10,100}. For RF, we performed a grid search over the number of trees (500, 1000, or 2000) and minimum node size (2 or 3). As SVM performs only binary classification, we used a one-versus-one strategy (i.e. we trained three models: Control vs. HRL, Control vs. CRC and HRL vs. CRC) to give a multi-class prediction. The whole setup was performed in parallel for the species, genus and family datasets.

## Results

### Taxonomy comparison

**Fig. 1:**
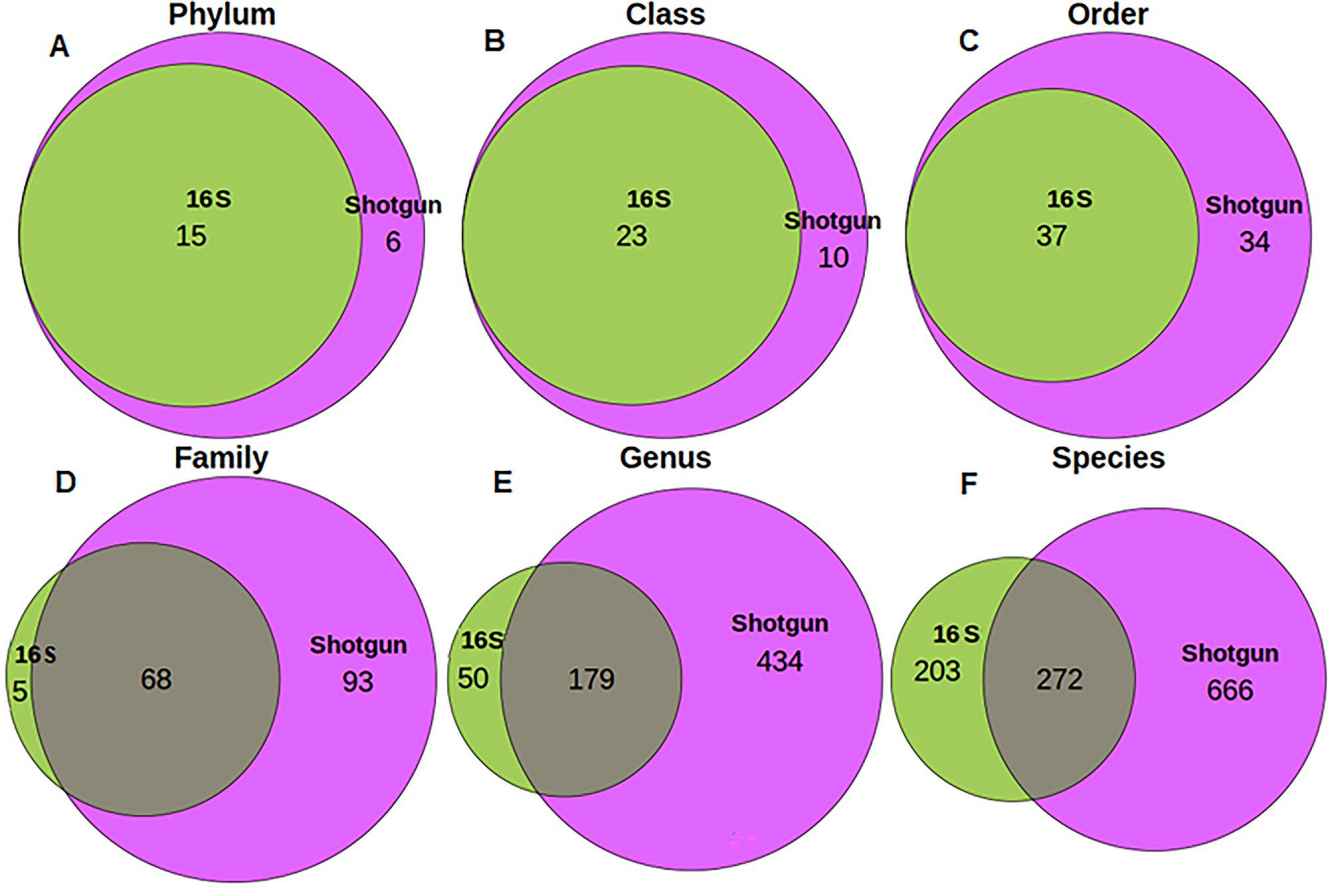
Venn diagrams of the common and non-common taxa of shotgun and 16S taking into account all samples at all taxonomic ranks

The median (range) sequencing reads of the 16S samples was 26,438 (ranging from 6,619 to 80,095) and 5,698,549 reads (from 2,813,748 to 397,298,642) for shotgun. Shotgun sequencing detected 4,512 different species belonging to 1,049 different genera and 214 families. Instead, 16S detected 525 species belonging to 239 genera and 80 families. We verified that rarefaction curves reached a plateau in terms of identified species, genera, and families (Additional Figure S1). Next, we computed the overlap between the taxa detected by 16S and shotgun. As expected, we found a great overlap in higher taxonomic ranks, where the 16S taxa is a subset of the shotgun taxa (Fig. 1). This changes in the family, genus, and species ranks. Overall, there were 15 phyla, 23 classes, 37 orders, 68 families, 179 genera, and 272 species that appear in both 16S and shotgun datasets (Fig. 1; Table 1). 6 phyla, 10 classes, 34 orders, 93 families, 434 genera and 666 species were present in shotgun samples but not in the 16S ones. Conversely, 5 families, 51 genera, and 203 species were present only in 16S sequenced samples but not in the shotgun ones. When analyzing sample by sample, the percentage of shared taxa was consistently lower than that observed in the overall comparison across all taxonomy levels (Table 1). In all scenarios, the lower the taxonomic rank, the lower the shotgun-16S agreement.

**Table 1:** Taxonomic agreement between shotgun and 16S. In the first column, number of unique taxa detected by 16S and shotgun sequencing methods, and shared taxa in absolute and relative frequency with respect to shotgun and 16S. all samples and taxonomic ranks are taken into account. In the second column, the 16S-shotgun agreement is computed per sample. We show the median and range of the shared taxa across all samples, the percentage of common taxa with respect to shotgun and 16S and Cohen’s Kappa agreement coefficient; and Spearman correlation coefficient (rho) median, quartile 1 (Q1) and (Q3)

Taxonomy level	16S	Shotgun	Shared	Shared/16S (%)	Shared/shotgun (%)	Shared Median (range)	Shared/16S(%) Median (range)	Shared/ shotgun(%) Median (range)	Rho Median (Q1, Q3)	Kappa Median (range)
Phylum	15	21	15	100.0	71.4	8 (4, 12)	100.0 (100.0, 100.0)	53.3 (26.7, 80.0)	0.95 (0.83, 0.99)	0.73 (0.20, 1)
Class	23	33	23	100.0	69.7	14 (7, 20)	100.0 (90.0, 100.0)	56.6 (28.8, 80.0)	0.89 (0.71, 0.95)	0.65 (0.30, 1)
Order	37	71	37	100.0	52.1	20 (9, 27)	100.0 (81.8, 100.0)	52.6 (23.1, 71.1)	0.90 (0.74, 0.96)	0.62 (0.13, 0.89)
Family	73	161	68	93.2	42.2	34 (12, 44)	94.1 (80.0, 100.0)	49.3 (17.6, 62.9)	0.76 (0.63, 0.88)	0.65 (0.03, 0.88)
Genus	229	613	179	78.2	29.2	70 (17, 97)	95.4 (88.6, 100.0)	39.5 (9.4, 54.5)	0.65 (0.45, 0.76)	0.50 (0.11, 0.70)
Species	609	938	355	58.3	37.8	114 (15, 175)	18.7 (2.5, 28.7)	12.2 (1.6, 18.7)	0.58 (0.38, 0.72)	0.47 (0.07, 0.62)

### Abundance comparison

**Fig. 2:**
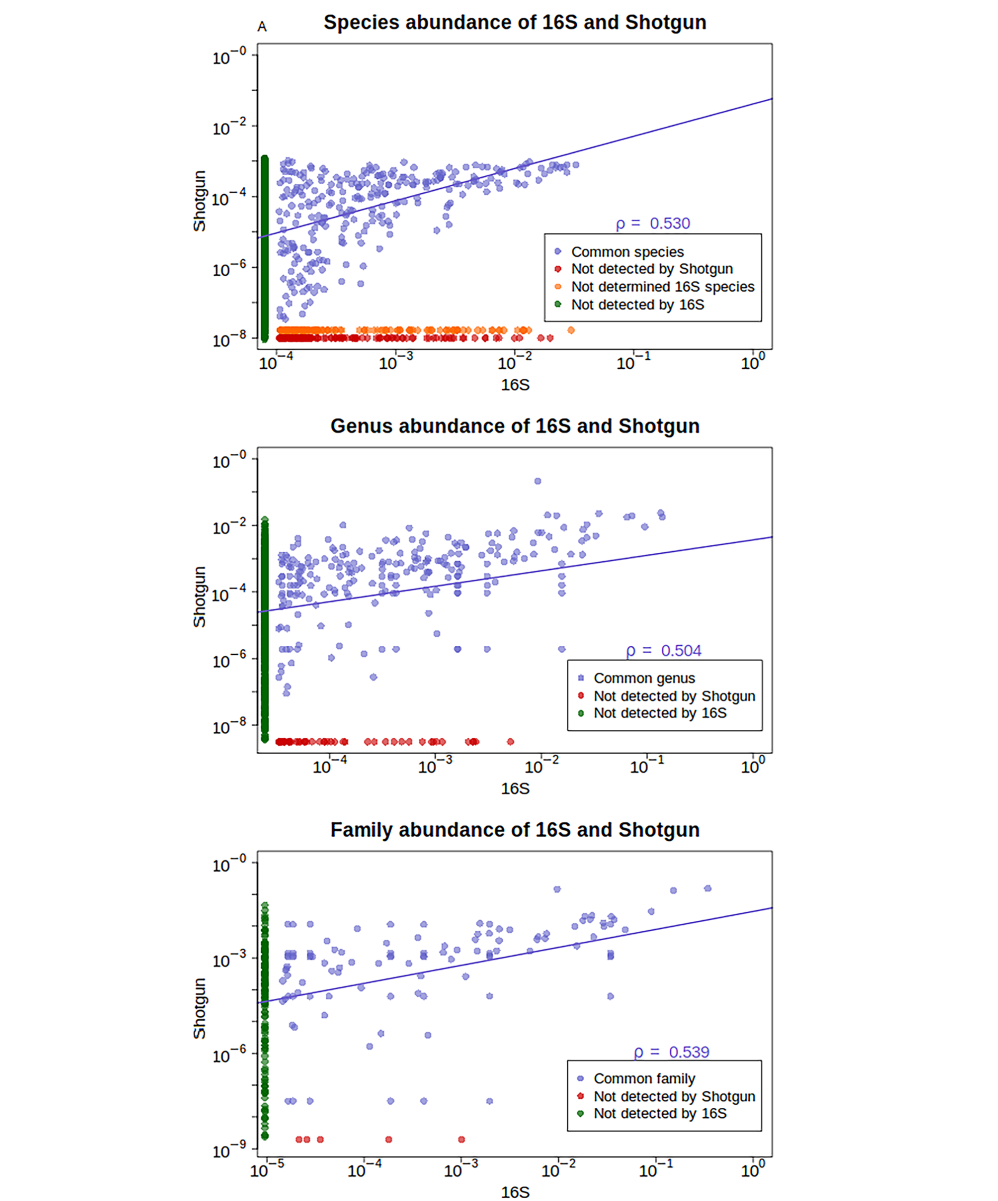
Decimal logarithm scatter plot representation of closed geometric means (centre of a composition) of Species, Genus and Families abundances in 16S and shotgun. The strength of the association over shared taxa is estimated with Spearman Correlation Coefficient and represented with a regression line. The blue dots indicate shared taxa, in green we can see the counts of the shotgun taxa that cannot be found in 16S, in red we can find the ones detected by 16S and not by shotgun. Furthermore, the unnamed 16S species are colored in orange

We found a positive correlation between the 16S and shotgun average counts of shared taxa within the Species, Genus, and Family taxonomic ranks (Fig. 2: Spearman correlation coefficient ranged from 0.50 to 0.53, *p*-values < 2e-16). We observed that the abundant species in 16S data also exhibited a high abundance in shotgun; however, the opposite was not always true: high-abundance shotgun species may appear in 16S in low frequencies. There were also some prevalent taxa according to one of the sequencing methodologies that were completely absent according to the other. Here we highlight some abundant taxa (> 64 counts per sample) in 16S that did not appear in the shotgun dataset: at the species level (Fig. 2a) *Coprococcus comes*, *Roseburia inulinivorans*, *Neglectibacter timonensis*, *Ruminococcoides bili*, *Erysipelotrichaceae UCG-003 bacterium*, *Phocaeicola vulgatus*, *Massiliprevotella massiliensis*, *Alistipes inops* and *Succinivibrio dextrinosolvens;* at the genus level (Fig. 2b) *Neglectibacter*, *Ruminococcoides*, *Erysipelotrichaceae UCG-003*, *Massiliprevotella*, *Mesorhizobium*, *Labrys*, *Escherichia-Shigella* and at the family level (Fig. 2c) *Erysipelatoclostridiaceae*, *Xanthobacteraceae.* When comparing abundance sample by sample, both the Spearman coefficient for counts and Cohen’s Kappa coefficient for binary variables revealed a substantial agreement between shotgun and 16S (Table 1). Specifically, the Spearman coefficient ranged from approximately 0.80 to 0.99 at higher taxonomy levels and from 0.38 to 0.72 at species level. Meanwhile, Cohen’s Kappa coefficient ranged from approximately 0.20–0.30 to 1 at higher taxonomy levels and from 0.07 to 0.62 at species level. To compute these coefficients, we exclusively used the 16S species that were unambiguous (i.e. without slashes).

Regarding the relative abundance of the shared 16S and shotgun taxa, we did not find major visual differences in most of the top 50 most prevalent species (Additional Figure S2a), genera (Additional Figure S2b) and families (Additional Figure S2c). Nevertheless, a few taxa showed differences between the sequencing methods. Some of them were *Prevotella copri*,* Eubacterium coprostanoligenes*,* Blautia massiliensis*,* Methanobrevibacter smithii* and *Holdemanella biformis* at the species level; *Collinsella*, *Streptococcus*, *Alistipes*, *Ruminococcus*, *Bacteroides*, and *Blautia* at genus level and *Bacteroidaceae*, *Methanobacteriaceae*, *Streptococcaceae*, and *Erysipelotrichaceae* at the family level, among others.

**Fig. 3:**
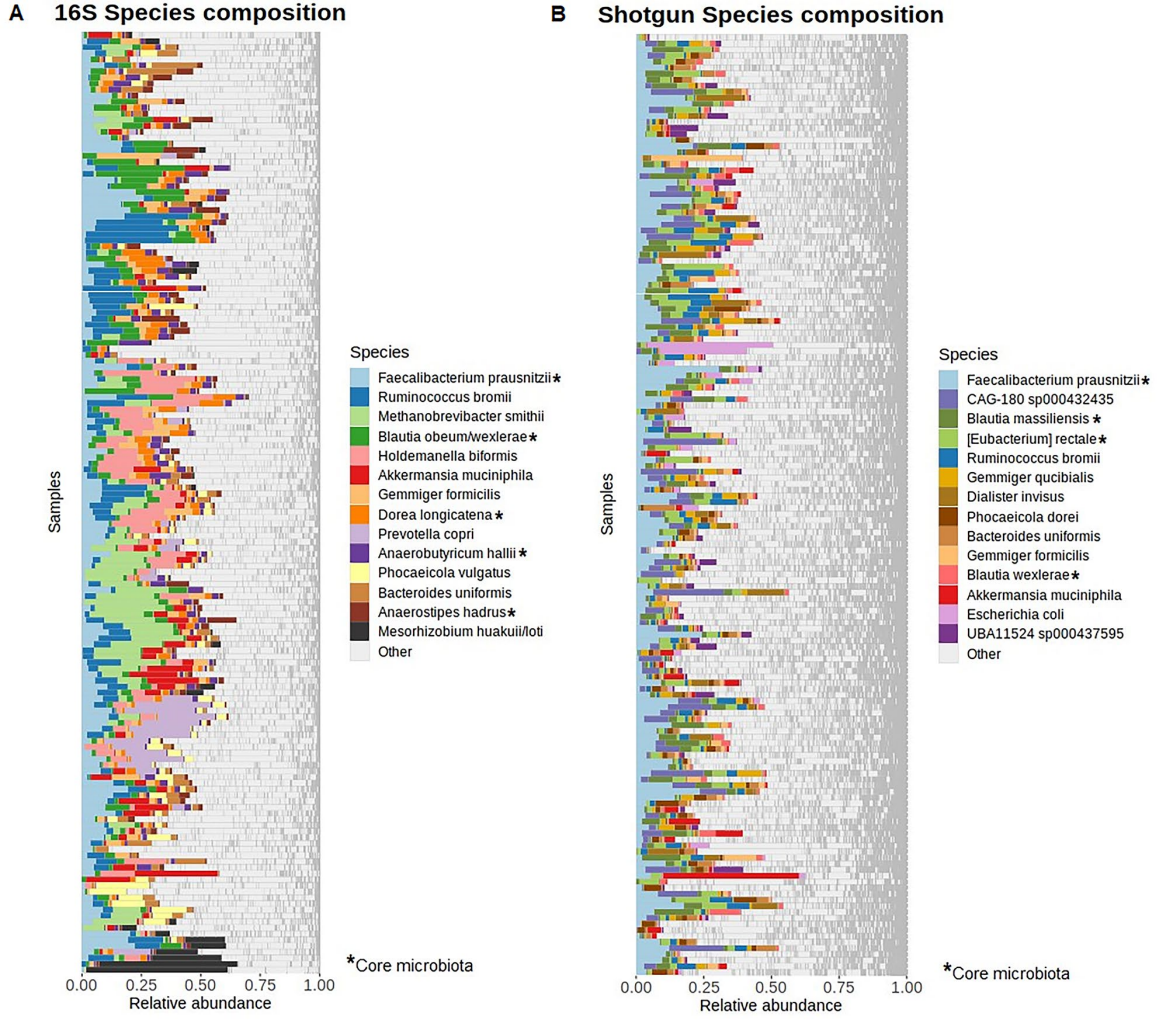
Bar plots of the top 15 most common species in 16S and shotgun abundance data. Each row represents a different sample with the same order in both plots

Finally, we compared the relative abundance of the top 15 most prevalent species between the two sequencing methods across the 156 samples (Fig. 3a and b). It can be observed that 16S samples were dominated by fewer microbial species than shotgun samples. The predominant species in both shotgun and 16S was *Faecalibacterium preausnitzii.* Other shared species in top 15 were: *Ruminococcus bromii*,* Akkermansia muciniphila*,* Gemmiger formicilis*,* Bacteroides uniformis* and *Blautia wexlerae.* Moreover, the top 3 genera were the same in both methods: *Blautia*,* Ruminococcus* and *Faecalibacterium* (Additional Figure S3a; Additional Figure S3b), though in a different order. The other genera in the top 15, like *Phocaeicola*,* Bacteroides*,* Bifidobacterium*, were shared by the two methods. Finally, *Lachnospiraceae*, *Oscillospiraceae* and *Bacteroidaceae* were in the top four families both in 16S and shotgun (Additional Figure S3c; Additional Figure S3d). The other shared families in the top 15 most prevalent were *Bifidobacteriaceae*, *Prevotellaceae*, *Eubacteriales Order* and *Enterobacteriaceae*.

### Sparsity, alpha and beta diversity

**Fig. 4:**
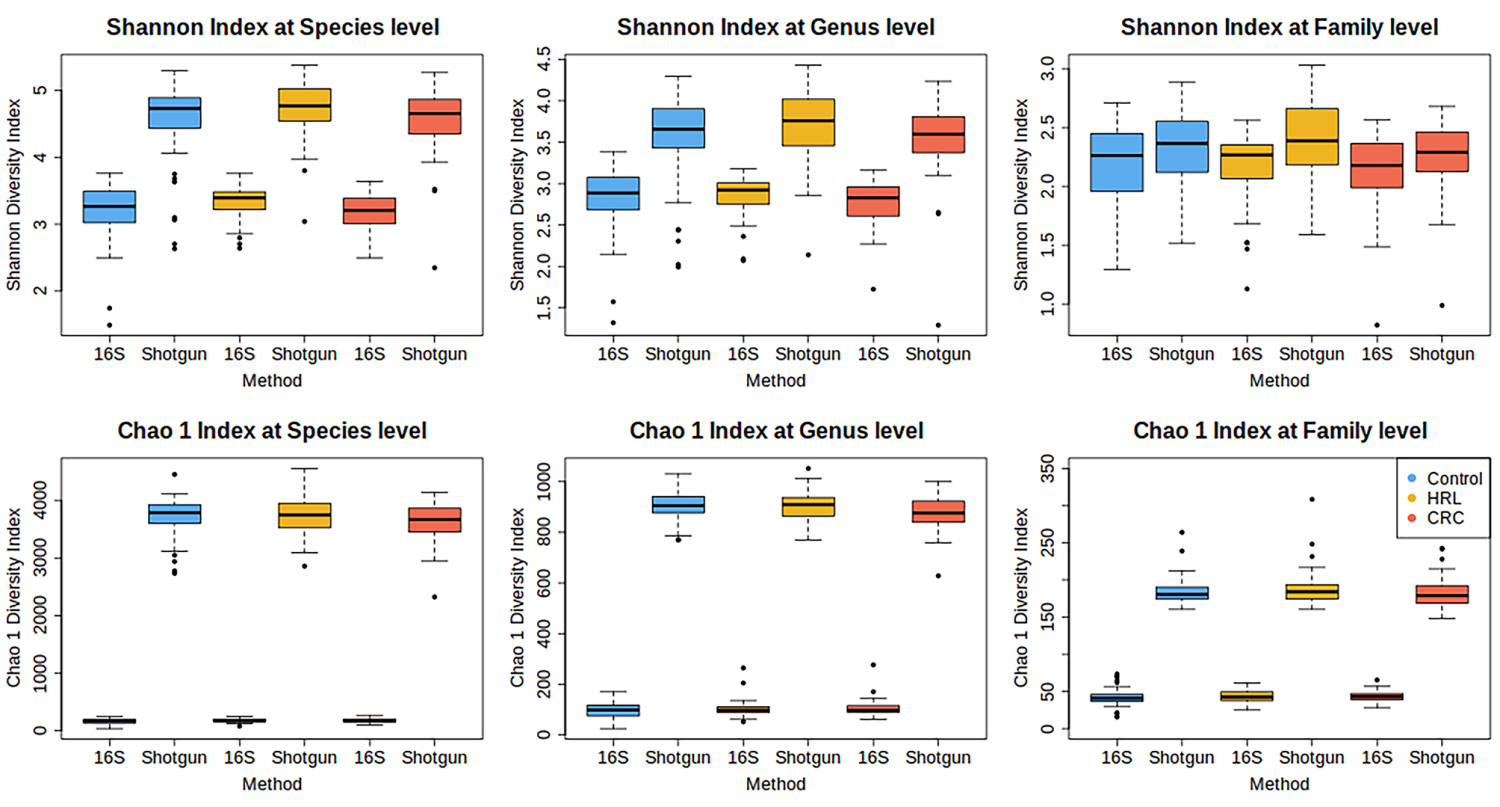
Box plots of the Shannon and Chao 1 Diversity Indexes for 16S and shotgun stratified per diagnosis (controls, HRL and CRC cases) at Species, Genus and Family taxonomy level

**Fig. 5:**
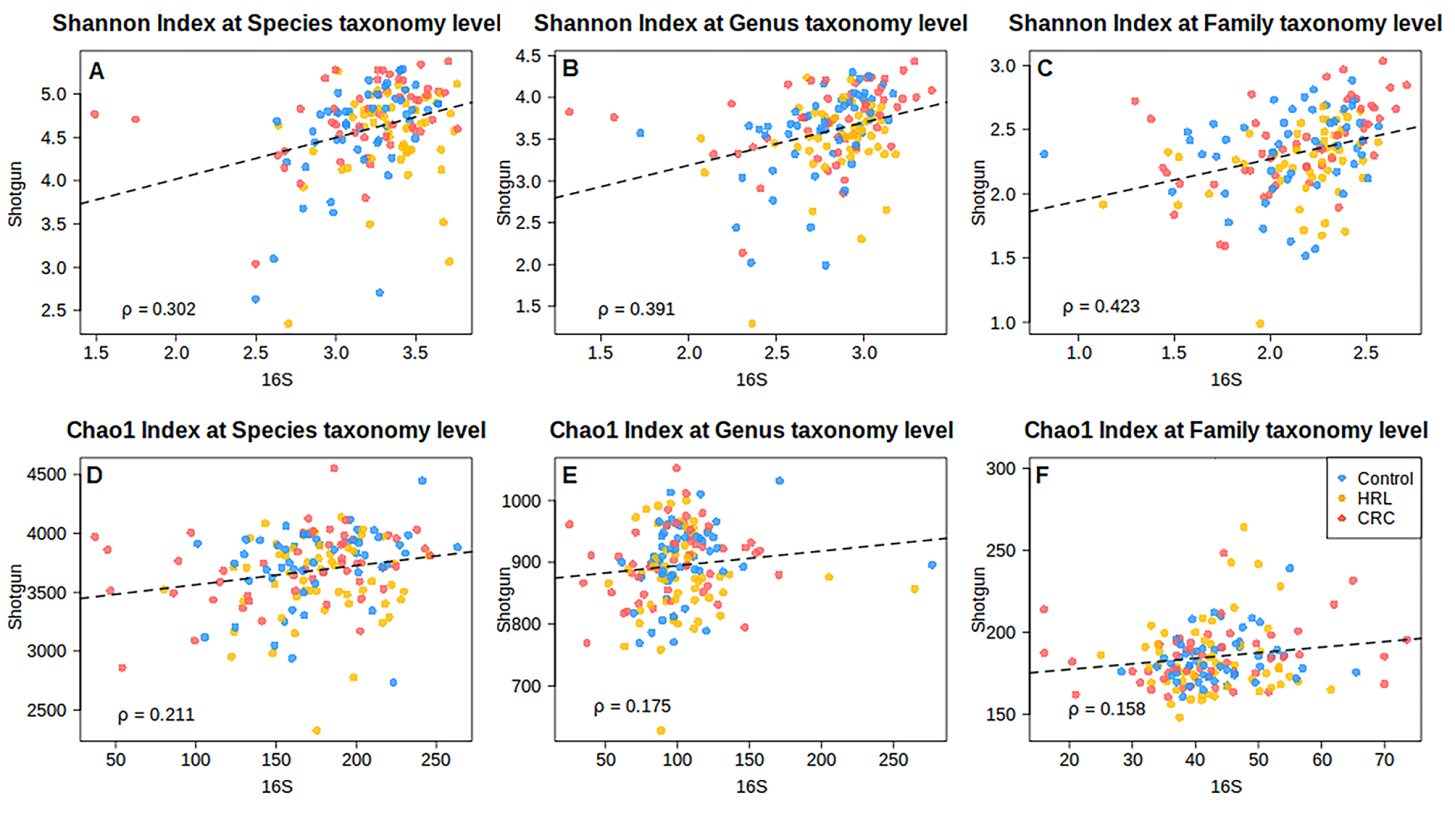
Scatter plot of the Shannon and Chao 1 Diversity indexes in 16S and shotgun colored by diagnostic status. The association is measured with Pearson Correlation Coefficient and represented with a dashed regression line

Both Shannon and Chao 1 diversity indices showed statistically significant differences between the two sequencing methods at the species, genus and family level (Fig. 4; Additional Table S1). Samples sequenced using shotgun had a statistically significant higher diversity than the 16S ones, especially in richness, estimated by Chao1. However, for the Shannon index, differences were smaller as we ascended from the species to the family level. When we computed the Spearman correlation between shotgun and 16S’ alpha-diversities, we found a positive correlation for both diversity indexes at the three taxonomy ranks (Fig. 5). Spearman correlation index ranged from 0.16 to 0.42, statistically different from 0 in all cases (*p*-values < 1e-4 for Shannon index and < 0.02 for Chao1). Specifically for the Shannon index, there was a slight increase in correlation values with the higher the taxonomic rank. We identified the outliers in the regression lines in Fig. 5, which can be attributed to technical issues. The two 16S CRC samples with lower alpha-diversity than their shotgun counterparts had very few reads. In fact, according to the rarefaction curves, they were the two 16S samples with the less observed species. The outlier HRL shotgun sample had 50% of *Escherichia coli* resulting in a notably low diversity (in its paired sample in 16S, *Escherichia coli/Shigella sonnei* account for 25% of the total abundance). This particular sample had the minimum coverage and species richness in the shotgun analysis.

**Fig. 6:**
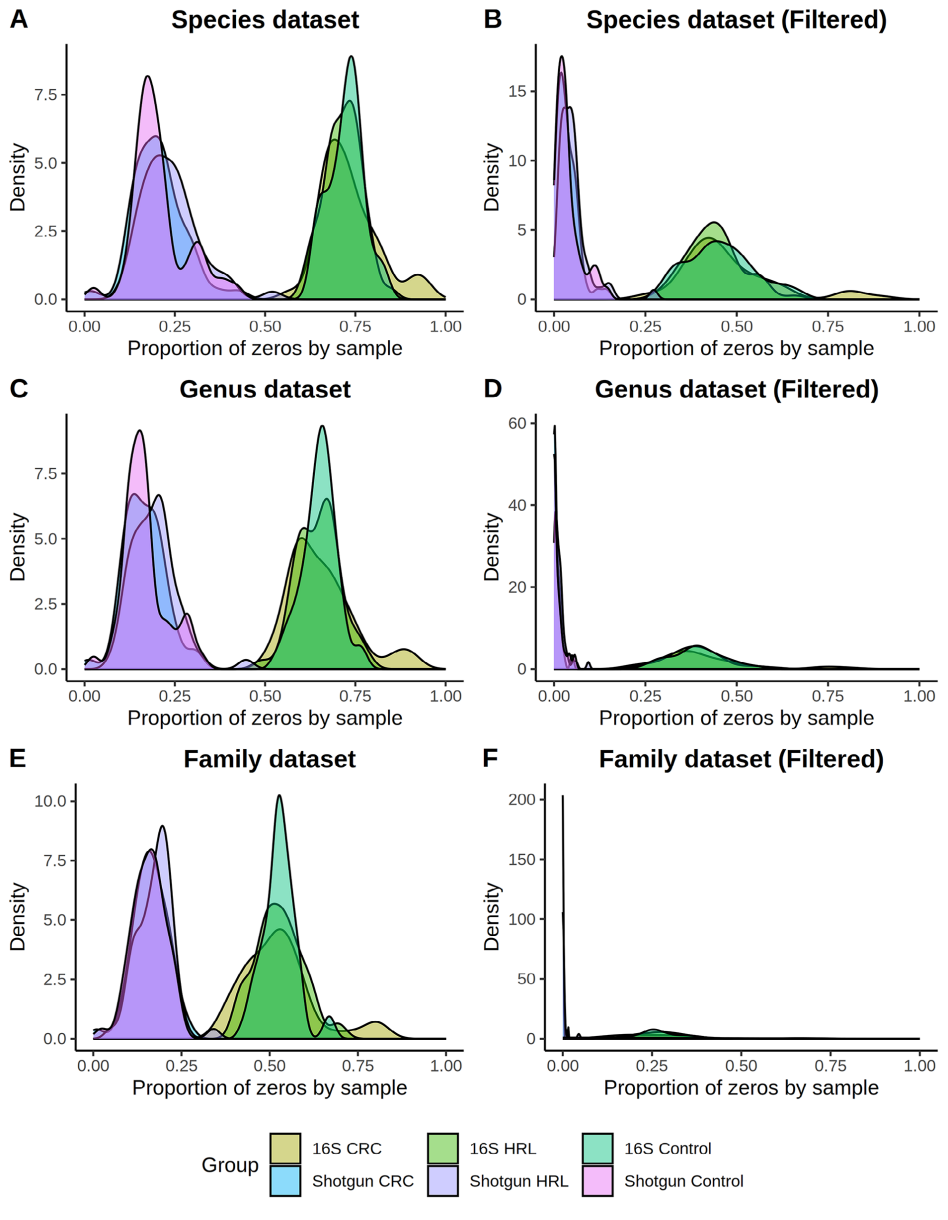
Shotgun vs. 16S sparsity. The zeros’ proportion of the controls, HRL and CRC cases in shotgun (blue-purple) and 16S (brown-green) abundance data. As the number of taxa differs between the two matrices, the proportion of zeros was computed by sample. Kruskal-Wallis among the three diagnostic groups is shown in Additional Table S2. Left panels correspond to unfiltered data, while right panels correspond to filtered data. First row is for the Species rank, second row is for Genus and third is for Family

The 16S count matrix was much sparser than shotgun’s (Wilcoxon Rank Sum Tests *p*-value < 2.2e-16), in the three studied ranks (family, genus, and species) before and after filtering rare taxa (Fig. 6). When comparing diagnosis, we could observe that in the 16S data controls, HRL and CRC samples presented similar sparsity levels (Additional Table S2). In the shotgun species and genera datasets, HRL were slightly sparser than the other two groups. However, these differences were no longer significant after Bonferroni correction (α = 0.05/12).

**Fig. 7:**
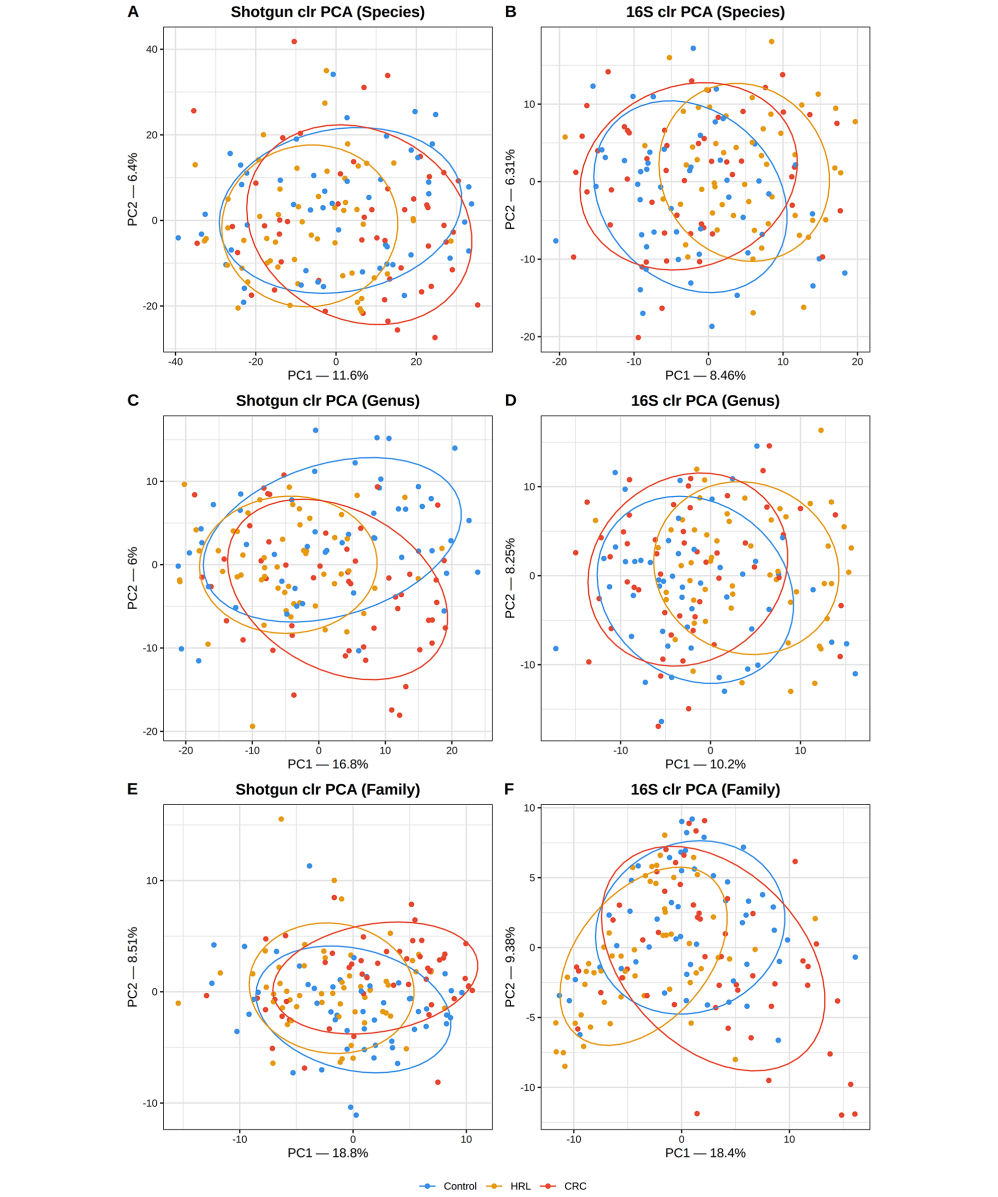
Shotgun and 16S Aitchison PCoAs. Only the two first axes are shown, along with their corresponding % of explained variance. Controls are in blue, HRL samples in yellow and CRC samples in red. Shotgun PCoAs are in the left panels, while 16S’ are in the right panels. First row is for the Species rank, second row is for Genus and third is for Family

From this point forwards we exclusively utilized the filtered datasets. After filtering, shotgun retained 466 species, 213 genera and 67 families, while 16S retained 191 species, 105 genera, and 44 families. Figure 7 shows the Aitchison distance PCoAs, representing the shotgun and 16S data for the three taxonomic ranks studied. There was a great overlap among the three diagnosis groups. In the shotgun PCoAs, HRL appeared as an intermediate group between controls and CRC cases. Instead, in the 16S PCoAs, HRL samples clustered aside from both controls and cases; this is especially striking in the Family clr-PCoA. Nevertheless, these were minor differences observed visually in the graph. It is important to note that the PCoA plot depicts only the first two principal components, and thus does not capture all the variability in the data. Additionally, we found extremely similar R statistic values when comparing the similarity of samples within groups respect to between groups for each method. At the species level, the ANOSIM R values were 0.08 for 16S and 0.07 for shotgun. At the genus level, the R values were 0.08 for 16S and 0.09 for shotgun. At the family level, the R values were 0.10 for both 16S and shotgun. Values larger than zero indicate differences among groups, which were confirmed by a PERMANOVA and its posthoc analysis with adjusted *p*-value < 0.05, irrespective of the sequencing technique and taxonomic rank (Additional Table S3).

Subsequently, Co-Inertia Analysis and Procrustes analysis were used to assess and compare the correlation between the Shotgun and 16S PCoAs. In all cases the permutation test gave *p*-values = 0.001, statistically different from 0. The co-inertia RV coefficient had the following values: Species = 0.52, Genus = 0.43 and Family = 0.35. Procrustes *r* had the following values: Species = 0.77, Genus = 0.64 and Family = 0.44. That is, both analyses of the PCoA projections showed a decreasing similarity pattern: the higher the rank, the lower the similarity. Finally, the relations between the 6 datasets (shotgun vs. 16S across 3 taxonomic ranks) were visually summarized using a PCA (Additional Figure S4). It is clearly evident that the primary difference among datasets is the sequencing technique (first PC: shotgun vs. 16S) followed by the aggregation of taxa from species to genus to family (second PC).

### Machine learning models

Finally, an assessment was conducted to determine the similarity between prediction models trained either on shotgun or 16S abundance data. Table 2, shows the accuracies for both training (i.e. the fitting) and test sets of the RF and SVM models (shotgun and 16S across 3 taxonomic ranks). Test accuracies were clearly lower than those of the training sets’, indicating a certain degree of overfitting, a common drawback when dealing with small training sets. 16S appeared to be more affected by overfitting than shotgun. Overall, SVM gave better results in shotgun and RF in 16S data, although it was difficult to make a conclusive statement as the accuracy 95% CIs were very wide due to the small size of the test. In fact, only two models achieved accuracies above the random model threshold and thus could be presumed to have some degree of predictive power: SVM shotgun Species = 56.7 (95%CI: 38.9, 74.4), and SVM shotgun Family = 53.3% (95%CI: 35.5, 71.2).

**Table 2:** Training and test accuracies (%) for the RF and SVM prediction models. Test 95%CI obtained via bootstrap (*n* = 2000). Performances above the random model (accuracy = 33.34%) are in **bold**

	Models	RF Fitted (%)	SVM Fitted (%)	RF test (%)	SVM test (%)
Species	Shotgun	54.0	75.4	43.3 (26.7, 60.0)	**56.7 (40.0**,** 73.3)**
	16S	61.9	80.2	43.3 (26.7, 60.0)	46.7 (30.0, 63.3)
Genus	Shotgun	50.8	69.8	36.7 (20.0, 53.3)	43.3 (26.7, 60.0)
	16S	54.8	74.6	50.0 (30.0, 66.7)	46.7 (30.0, 63.3)
Family	Shotgun	47.6	69.1	40.0 (23.3, 56.7)	**53.3 (36.7**,** 70.0)**
	16S	52.4	69.1	50.0 (30.0, 66.7)	40.0 (23.3, 56.7)

Next, the agreement between shotgun and 16S models built from data of the same taxonomic rank was assessed. Cohen’s kappa and its 95% CI was also computed, and both results are presented in Table 3. Overall, the agreement was lower in the RF models, which can be attributed to the random nature of the RF algorithm. The fitted models presented a fair-moderate agreement (∼ 0.37) over the training data. However, agreement in the test was much lower, which is consistent with the overfitting we observed in Table 2. Likewise, wide 95%CIs were observed and the only Cohen’s kappa not including zero belonged to the SVM models over species data: 0.33 (95%CI: 0.05, 0.60).

**Table 3:** Training and test classification coincidence between the shotgun and 16S models: proportion of agreement (between 0 and 1) and Cohen’s Kappa. Statistically significant Cohen’s kappa are highlighted in **bold**

	Models	Agreement		Cohen’s Kappa	
		Training	Test	Training	Test
Species	RF	0.60	0.43	**0.39 (0.25**,** 0.52)**	0.14 (-0.11, 0.38)
	SVM	0.71	0.57	**0.55 (0.42**,** 0.67)**	**0.33 (0.05**,** 0.60)**
Genus	RF	0.47	0.30	**0.19 (0.06**,** 0.32)**	-0.10 (-0.31, 0.11)
	SVM	0.63	0.50	**0.43 (0.30**,** 0.55)**	0.15 (-0.11, 0.41)
Family	RF	0.53	0.40	**0.30 (0.16**,** 0.43)**	0.11 (-0.10, 0.33)
	SVM	0.61	0.53	**0.38 (0.25**,** 0.52)**	0.19 (-0.09, 0.47)

The SVM classification models are binary by nature, and so the SVM model presented earlier was an ensemble of the three two-group models (control vs. HRL, control vs. CRC and HRL vs. CRC). In Additional Table S4, the agreement results are presented separately for these three classifiers. It can be observed that the aforementioned high Cohen’s Kappa was driven by a substantial concordance between shotgun and 16S control vs. CRC models: Cohen’s kappa = 0.47 (0.14, 0.80). Instead, in higher taxonomic ranks, control vs. HRL and HRL vs. CRC are the most concordant. Following our exploration of the SVM models, Additional Table S5 shows the concordance between models with regard to their Support Vectors (SV), i.e. the training patients each model considers relevant to discriminate between the classes. In the three taxonomic ranks, the control vs. CRC models were the more akin with regard to their SV, followed by the HRL vs. CRC models.

**Fig. 8:**
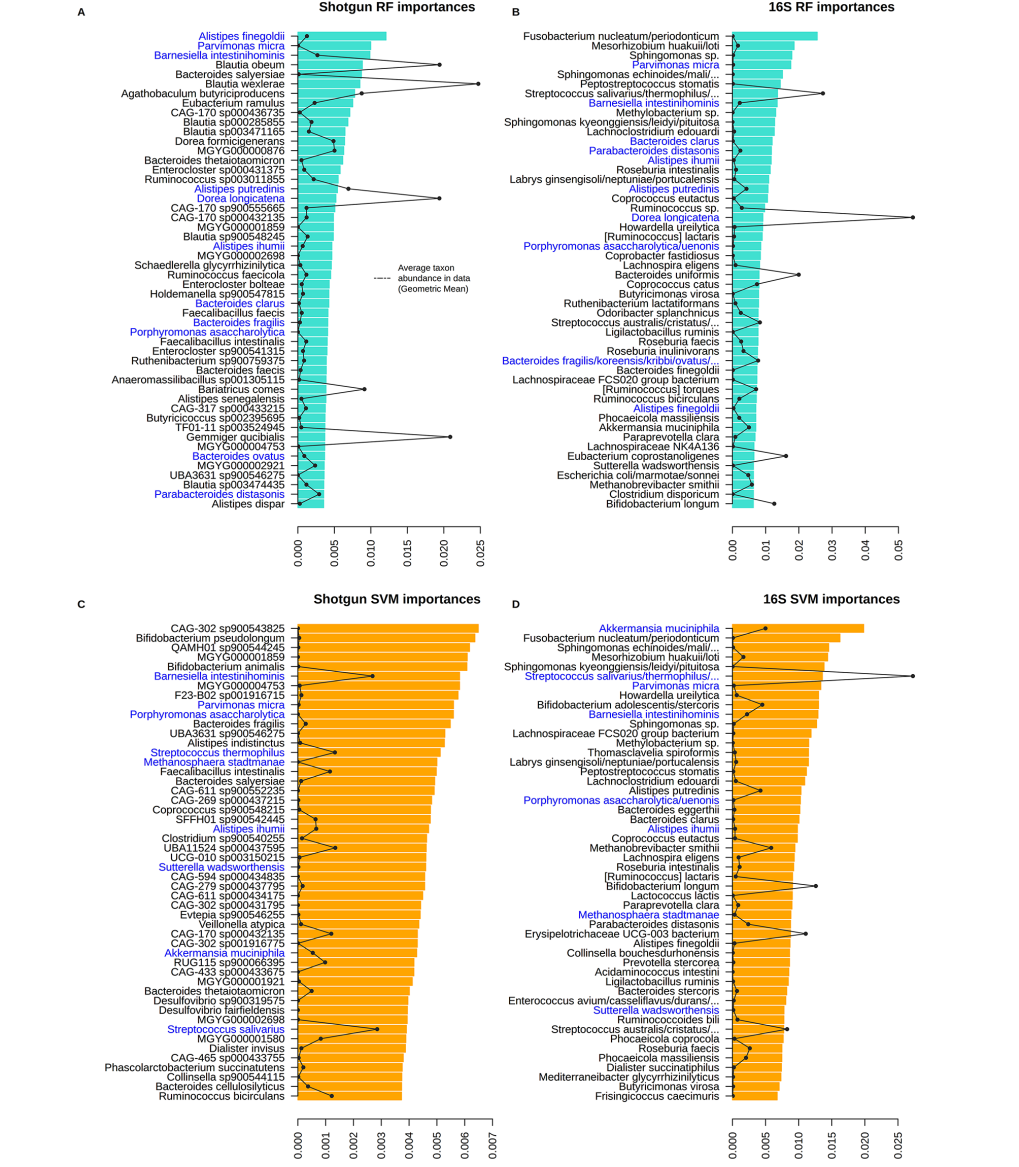
Microbial signature agreement. Barplot represents the top-50 importances of the Species models, sorted. RF importances are in turquoise and SVM importances in orange. Left panels correspond to the Shotgun data, while right panels are for the 16S data. Species common to 16S and shotgun signatures are highlighted in blue. Note: Complete names of the 16S taxa that have been truncated in the plot: • *Sphingomonas echinoides/mali/oligophenolica/sanxanigenens* • *Streptococcus salivarius/thermophilus/vestibularis* • *Streptococcus australis/cristatus/gordonii/infantis/mitis/oralis/parasanguinis/pneumoniae/pseudopneumoniae/sanguinis* • *Bacteroides fragilis/koreensis/kribbi/ovatus/xylanisolvens* • *Enterococcus avium/casseliflavus/durans/faecium/gallinarum/hermanniensis/hirae/hiraes/phoeniculicola/raffinosus/saccharolyticus/thailandicu*

Finally, we studied the similarity between “microbial signatures” (the taxa considered most important by a model to perform the prediction). The complete list of shotgun and 16S taxa, ranked by importance, were compared with the Kendall rank correlation coefficient (Table 4) The strength of Kendall correlation between shotgun and 16S was fair-moderate (around 0.22–0.31) and did not show great differences across taxonomic rank or ML method (RF, SVM). The top-50 species for the shotgun and 16S SVM and RF models are presented in Fig. 8, while the equivalent plot for Genus and Family models can be viewed in Additional Figures S5 and S6. In the Species models, we can find several taxa that have been previously related to the ACS (Parvimonas micra,* Bacteroides fragilis*,* Streptococcus thermophilus* or *Fusobacterium nucleatum*), and their lineages were also present in the genus and family signatures. Some of these CRC-related species (e.g., *Parvimonas micra*) were common to shotgun and 16S models. For clarity, Fig. 8 highlights all taxa shared by the shotgun and 16S, while Table 4 details the percentage of agreement between signatures. In RF models, there was at least 22% of coincidence between the top-50 taxa, while in SVM there was at least 18% (as a fraction of the taxa is unnamed in 16S and/or shotgun databases, real agreement may be greater). If we compare these species’ signatures counting the genera they share, both RF and SVM showed an agreement of 36%. Thus, we can consider that the “true” agreement is bounded by these two limits. Similar agreements are by the genus and family signatures. Additional Figure S7 provides the signature of the SVM binary models. Again, the highest coincidence was found in the control vs. CRC model, with agreement ranging from 22 to 28%.

**Table 4:** Taxa importance coincidence between the shotgun and 16S models: agreement and Kendall’s tau. Kendall rank correlation coefficient is computed considering all shared taxa in shotgun and 16S models. Agreement refers to the coincident taxa of the microbial signature shown in Fig. 8 (top-50 species), additional figures S5 (top 20 genera) and S6 (top 10 families). *p*-values < 0.05 are in **bold**

	Models	Agreement (top importances)	Kendall’s tau	
			Kendall’s tau (95%CI)	*p*-value
Species	RF	22–36%	**0.22 (0.05**,** 0.38)**	**4e-3**
	SVM	18–36%	**0.27 (0.13**,** 0.40)**	**5e-4**
Genus	RF	30%	**0.28 (0.14**,** 0.42)**	**4e-4**
	SVM	20%	**0.31 (0.17**,** 0.45)**	**1e-4**
Family	RF	30%	0.30 (0.03,0.57)	0.01
	SVM	30%	0.31 (0.11, 0.51)	0.01

## Discussion

Increasing interest in the role of the gut human microbiome during CRC development raises the urgency of selecting appropriate methods for unraveling human-associated microbial communities. Taxonomic resolution has been widely cited as one of the main differences between shotgun and 16S [16, 22, 26, 44, 45]. This difference impacts not only the “depth” of the lineages they are able to assign (16S datasets are often limited to the genus level) but their ability to represent the broad picture of a microbial community, capturing even rare taxa. On the other hand, this attention to detail has an impact over abundance data. Moreover, using different reference databases for 16S and shotgun sequencing can introduce inconsistencies. To address this issue, we updated all taxonomic lineages obtained for 16S and shotgun analyses using two specific R packages related to NCBI (package *myTAI* and *taxonomizr*), see Material and Methods for further details.

In this study we have followed a compositional approach, i.e. we have studied the relative frequencies of microorganisms belonging to a community– in fact, absolute frequencies in this kind of data may be not informative [35]. In this scenario a change in any of the parts comes at the expense of the rest: when a new bacterium is detected in a sample, the relative abundance of some or all the other decreases. Even without any primer or database bias, this dependency may cause that some taxa appears inflated when comparing 16S to shotgun [46]. Also, this suggest that shotgun needs a very good coverage (some studies recommend > 500,000 reads [26]) not only in order to detect rare taxa, but also to have enough reads to give reliable abundance estimations.

With a very good coverage, our results support that shotgun provides a more detailed resolution of the gut microbial community than 16S. It detected many more species (9x in our data), including rare species, and consequently had greater levels of alpha diversity. Instead, 16S data were much sparser, even after filtering by infrequent taxa. This sparsity, as well as the visual representation of the most frequent species (Fig. 3), proves that 16S samples are dominated by fewer bacteria. This is likely not due to sequencing depth, as rarefaction curves arrived at the plateau. All these findings are in line with most of the previous literature [16, 22, 25, 26, 44]. Only a few studies arrived at different conclusions: see, for instance Tessler et al., 2017 and Zuo et al., 2022 stated that 16S yielded more diverse bacterial phyla and families than shotgun sequencing, but the reference database lacked reference sequences for their data, and the available reads they had for taxonomic profiling were low (approx. 360,000) [21, 26], which could explain their findings. On the other hand, Zuo et al., 2022 found that shotgun and 16S provided a similar alpha-diversity in their samples.

Taxonomically speaking we found that the lower the taxonomic rank, the lower the shotgun-16S agreement, and this holds both when considering all organisms detected (i.e. columns of the abundance table) and when computing the percentage of shared taxa per sample. In higher ranks (phylum to order), l6S taxa were a mere subset of shotgun. It is noteworthy that almost 25% of the genera detected by 16S were not detected by the shotgun strategy. Upon investigating this issue, we found that the vast majority of genera detected by 16S but not shotgun are human gut inhabitants not included in the UHGG v2.0.1 database: indicating an issue of “closed annotation” [14]. Furthermore, when both 16S and shotgun detected a taxon, a moderate to substantial correlation was observed in the counts identified by these two sequencing technologies. This correlation was evident not only on average but also when computed sample by sample. In Results we highlighted some taxa that presented more than 64 counts for 16S but were undetected by shotgun. Again, most of them are compatible with the human gut but are not included in the UHGG v2.0.1 database [34]. Nevertheless, there are some exceptions: the ambiguous 16S genus *Escherichia/Shigella* (*Escherichia* is present in shotgun samples, but not *Shigella*) and genera *Mesorhizobium* and *Labrys* (from the family *Xanthobacteraceae*). The latter bacteria are usually found in soil, which may point to a possible contamination or a misidentification by the reference database. For a detailed description of this issue for the 50 genera only detected on 16S, see Additional File S1. It should be noted that, contrary to UHGG, the 16S reference database (SILVA) is not specific to the human gut.

Regarding diversity measures, shotgun had substantially higher diversity than 16S. A moderate, positive correlation between these two sequencing methods was observed for the Shannon index. This correlation increased at higher taxonomic ranks: at the family level, 16S samples were more similar to shotgun in evenness/richness (Figs. 4 and 5; see also Fig. 3). Unsurprisingly, the correlation was low for the Chao1 index, which includes information on the richness of very rare taxa. As for beta-diversity comparisons, neither sequencing method delivered clear-cut clusters of microbial community structure across the three diagnostic groups in PCoA (though PERMANOVA confirmed that differences existed). However, shotgun placed the HRL samples between the controls and CRC cases (which would be consistent with considering these lesions an intermediate entity between health and cancer), while 16S tended to separate them into a third group. Despite Co-inertia and Procrustes analyses showing a substantial correlation in community structure between shotgun and 16S, one of the most intriguing findings in our results was the large dissimilarity in community at higher the taxonomic ranks. This observation contradicted our hypothesis and diverged from some other metrics shown in this paper. Consistent to our findings, a similar pattern was observed by Tessler et al., 2017. We hypothesize that this may be caused by decreasing the dimensionality, so a small abundance difference in one taxon may significantly impact the resulting ordination more than when working with the higher-dimension species dataset.

We have discussed above the main taxonomic and abundance divergences between shotgun and 16S in our samples. However, a second factor enters into play when training and comparing ML models for diagnostic prediction. These models demand abundance differences in the three groups of enough magnitude to make them separable; also, these differences should be strong enough to be observed both in shotgun and 16S. There is abundant literature that shows that ML models have a good performance in discriminating healthy from CRC and HRL microbial samples [7, 9–12, 14, 47]. Only a few authors contrasted shotgun versus 16S models [9, 29], either noting that they yielded similar performances or that shotgun was slightly better than 16S. Although the small training size hinders our models, our results are consistent with previous literature on this point. In addition, we also studied the agreement between shotgun and 16S models regarding their fitted and predicted labels and their SV. We found the maximum agreement in the SVM species model, and this agreement seems be driven mainly by the control vs. CRC classification. If we consider controls and CRC cases as extremes of a continuum, it is logical that the greater differences (which should be easier to observe) arise when comparing the extremes. Moreover, it has been consistently reported higher performances classifying control vs. CRC than control vs. HRL [10, 13, 14]. On the other hand, our 16S models tended to consider that HRL have a distinct microbial pattern. This is consistent with the PCoAs (Fig. 7) and with other 16S studies that found that the CRC microbial markers are specific and have limited value to predict HRL [14].

Regarding microbial signatures, we detected some species previously linked to CRC that show a moderate correlation between shotgun and 16S. As discussed before, the predictive importance of a taxon is completely unrelated to its prevalence (Fig. 8; see also Fig. 3 and Additional Figure S2). Among the most frequent species in our 16S and shotgun samples, only *Akkermansia muciniphila* appears in both microbial signatures and it has been associated previously to CRC [32]. However, some low-abundance bacteria that are very discriminative of the phenotype of interest may be sidelined during pre-processing. This is the case of *Fusobacterium nucleatum*, a key species in CRC progression, which appears as the 1st (RF) or 2nd (SVM) most important species in the 16S microbial signature (Fig. 8), but did not pass the shotgun’s filtering step. Shotgun genus-level models include *Fusobacterium* in their microbial signature (Additional Figure S5). On the other hand, the lower taxonomic resolution of 16S led to ASV ambiguity that probably hindered the prediction models. For instance, shotgun models highlighted the CRC-enriched *Bacteroides fragilis* and control-enriched *Bacteroides ovatus* [46], which appeared in 16S models as a single feature *(Bacteroides fragilis/koreensis/kribbi/ovatus/xylanisolvens)* and with lower importance. In any case, the high consistency in shotgun and 16S of some other CRC microbial biomarkers like *Parvimonas micra* [7, 10, 11, 13, 14] and *Porphyromonas asaccharolytica* [9–11] is remarkable. Regarding HRL status, as mentioned by Thomas et al., 2019 and Obón-Santacana et al., 2022, it is an arduous task to find some microbiome markers at this stage. Taking into account this increased difficulty, along with the scarce literature available on the genus *Alistipes* in relation to HRL and CRC, the SVM results suggested *Alistipes ihumii* as a possible candidate for an HRL biomarker.

BesiBesidess, our models showed that a significant number of species associated with a healthy human gut were found in common between the two compared techniques. These species were: *Barnesiella intestinihominis* [48], *Bifidobacterium longum* [49], *Methanobrevibacter smithii* [50], *Sutterella wadsworthensis* [51], and *Ligilactobacillus ruminis* [52].

### Study limitations

A primary limitation of our study is the relatively modest sample size. Though the sample size probably is enough for our main aim, the comparison between 16S and shotgun diversity, and composition, it is insufficient for the comparison of CRC and HRL prediction models. A larger sample size would have favored narrower CIs and prevent ML overfitting. Another important challenge was the fraction of ambiguous (in 16S) or unnamed species (especially prevalent in shotgun), which we were forced to exclude to enable a taxonomic comparison between shotgun and 16S. In fact, we have proven with different examples that part of the differences between both sequencing methods are related to differences between the reference databases. Therefore, the issue of “closed annotation” of the taxonomical databases imposes a strong limitation when comparing shotgun with 16S. Recently, a new version of the Greengenes database appeared (Greengenes2) [53] that provides a single reference database for both sequencing technologies, which could aid in this up-to-date limitation of having independent databases. Currently, the Greengenes2 is available in Qiime2 pipeline for 16S and in the Woltka for shotgun. Surely this update will be available in the near future in other commonly used taxonomical classifiers (for example, DADA2 for 16S and Kraken2 for shotgun, which currently offers the older Greengenes database). On the other hand, despite involving the same participants, the 16S and shotgun data were obtained at different times. As explained in the Methodology, different DNA extraction kits were used, and sequencing was conducted by two different companies. This was required by the service contracting policies that govern our institution.

## Conclusions

Shotgun and 16S sequencing provide two different lenses to examine microbial communities. While we have demonstrated that they can unravel common patterns (including microbial signatures), shotgun often gives a more detailed snapshot than 16S, both in depth (lineage resolution) and breadth (number of species, including rare species). However, a downside to its high richness is that some (or all) taxa may appear in lower relative frequencies within a sample than expected. Researchers should be careful in this scenario, as high-interest bacteria may be filtered for having “low” abundance. On the other hand, 16S will tend to show only part of the picture, giving greater weight to dominant bacteria in a sample. Sometimes, bacteria of closely related lineages but opposing biological roles may be aggregated due to the lower taxonomic resolution. However, in cases where only representative bacteria are of interest, 16S may beat the shotgun approach. Furthermore, 16S reference databases have been curated for a longer time and may contain lineages not yet present in shotgun databases. Our comparative analysis reveals that shotgun sequencing exhibits slightly higher sensitivity and specificity in detecting CRC and HRL compared to 16S, attributed to its superior resolution and ability to comprehensively profile microbial functions and potential biomarkers. This makes it particularly well-suited for studies requiring precise microbial characterization, especially in stool microbiome samples. However, for tissue samples or studies with targeted aims, 16S remains a preferable option due to its efficiency and suitability for lower biomass samples. In summary, the choice between shotgun and 16S sequencing should be guided by the specific goals of the study, the type of samples being analysed, the required resolution and depth of microbial analysis, and the available resources. For comprehensive analyses with higher interpretability, particularly in stool samples, shotgun sequencing is recommended. Conversely, for studies with clear and targeted aims, 16S is a suitable choice.

### Electronic supplementary material

Below is the link to the electronic supplementary material.

Supplementary Material 1

Supplementary Material 2

Supplementary Material 3

Supplementary Material 4

Supplementary Material 5

Supplementary Material 6

Supplementary Material 7

Supplementary Material 8

Supplementary Material 9

Supplementary Material 10

Supplementary Material 11

Supplementary Material 12

Supplementary Material 13

## Data Availability

16S rRNA raw data available in the European Nucleotide Archive (ENA) under the project PRJEB71787. Shotgun raw data available in the European Genome-Phenome Archive (EGA) under the study ID EGAS00001007025. 16S rRNA and shotgun abundance tables, metadata and their R script codes used for the present work are available in the Zenodo repository: https://zenodo.org/records/12545235.

## References

[1] Siegel RL, Wagle NS, Cercek A, Smith RA, Jemal A. Colorectal cancer statistics, 2023. CA: A Cancer. J Clin. 2023;73(3):233–54. 10.3322/caac.21772 36856579

[2] Fischer J, Walker LC, Robinson BA, Frizelle FA, Church JM, Eglinton TW. Clinical implications of the genetics of sporadic colorectal cancer. ANZ J Surg. 2019;89(10):1224–9. 30919552 10.1111/ans.15074

[3] Alves Martins BA, de Bulhões GF, Cavalcanti IN, Martins MM, de Oliveira PG, Martins AMA. Biomarkers in colorectal cancer: the role of translational proteomics research. Front Oncol. 2019;9:1284. 31828035 10.3389/fonc.2019.01284 PMC6890575

[4] Housini M, Dariya B, Ahmed N, Stevens A, Fiadjoe H, Nagaraju GP, et al. Colorectal cancer: genetic alterations, novel biomarkers, current therapeutic strategies and clinical trials. Gene. 2024;892:147857. 37783294 10.1016/j.gene.2023.147857 PMC12237584

[5] White MT, Sears CL. The microbial landscape of colorectal cancer. Nat Rev Microbiol. 2023 [cited 2023 Nov 17]; 10.1038/s41579-023-00973-4 10.1038/s41579-023-00973-4 37794172

[6] Wong CC, Yu J. Gut microbiota in colorectal cancer development and therapy. Nat Rev Clin Oncol. 2023;20(7):429–52. 37169888 10.1038/s41571-023-00766-x

[7] Yachida S, Mizutani S, Shiroma H, Shiba S, Nakajima T, Sakamoto T, et al. Metagenomic and metabolomic analyses reveal distinct stage-specific phenotypes of the gut microbiota in colorectal cancer. Nat Med. 2019;25(6):968–76. 31171880 10.1038/s41591-019-0458-7

[8] Mizutani S, Yamada T, Yachida S. Significance of the gut microbiome in multistep colorectal carcinogenesis. Cancer Sci. 2020;111(3):766–73. 31910311 10.1111/cas.14298 PMC7060472

[9] Zeller G, Tap J, Voigt AY, Sunagawa S, Kultima JR, Costea PI, et al. Potential of fecal microbiota for early-stage detection of colorectal cancer. Mol Syst Biol. 2014;10(11):766. 25432777 10.15252/msb.20145645 PMC4299606

[10] Thomas AM, Manghi P, Asnicar F, Pasolli E, Armanini F, Zolfo M, et al. Metagenomic analysis of colorectal cancer datasets identifies cross-cohort microbial diagnostic signatures and a link with choline degradation. Nat Med. 2019;25(4):667–78. 30936548 10.1038/s41591-019-0405-7 PMC9533319

[11] Wirbel J, Pyl PT, Kartal E, Zych K, Kashani A, Milanese A, et al. Meta-analysis of fecal metagenomes reveals global microbial signatures that are specific for colorectal cancer. Nat Med. 2019;25(4):679–89. 30936547 10.1038/s41591-019-0406-6 PMC7984229

[12] Gao Y, Zhu Z, Sun F. Increasing prediction performance of colorectal cancer disease status using random forests classification based on metagenomic shotgun sequencing data. Synth Syst Biotechnol. 2022;7(1):574–85. 35155839 10.1016/j.synbio.2022.01.005 PMC8801753

[13] Obón-Santacana M, Mas-Lloret J, Bars-Cortina D, Criado-Mesas L, Carreras-Torres R, Díez-Villanueva A, et al. Meta-analysis and validation of a colorectal cancer risk prediction model using deep sequenced fecal metagenomes. Cancers (Basel). 2022;14(17):4214. 36077748 10.3390/cancers14174214 PMC9454621

[14] Wu Z, Hullings AG, Ghanbari R, Etemadi A, Wan Y, Zhu B, et al. Comparison of fecal and oral collection methods for studies of the human microbiota in two Iranian cohorts. BMC Microbiol. 2021;21(1):324. 34809575 10.1186/s12866-021-02387-9 PMC8607576

[15] Lauber CL, Zhou N, Gordon JI, Knight R, Fierer N. Effect of storage conditions on the assessment of bacterial community structure in soil and human-associated samples. FEMS Microbiol Lett. 2010;307(1):80–6. 20412303 10.1111/j.1574-6968.2010.01965.x PMC3148093

[16] Brumfield KD, Huq A, Colwell RR, Olds JL, Leddy MB. Microbial resolution of whole genome shotgun and 16S amplicon metagenomic sequencing using publicly available NEON data. PLoS ONE. 2020;15(2):e0228899. 32053657 10.1371/journal.pone.0228899 PMC7018008

[17] Chakravorty S, Helb D, Burday M, Connell N, Alland D. A detailed analysis of 16S ribosomal RNA gene segments for the diagnosis of pathogenic bacteria. J Microbiol Methods. 2007;69(2):330–9. 17391789 10.1016/j.mimet.2007.02.005 PMC2562909

[18] Wensel CR, Pluznick JL, Salzberg SL, Sears CL. Next-generation sequencing: insights to advance clinical investigations of the microbiome. J Clin Invest. 2022;132(7):e154944. 35362479 10.1172/JCI154944 PMC8970668

[19] Hilton SK, Castro-Nallar E, Pérez-Losada M, Toma I, McCaffrey TA, Hoffman EP, et al. Metataxonomic and metagenomic approaches vs. culture-based techniques for clinical pathology. Front Microbiol. 2016;7:484. 27092134 10.3389/fmicb.2016.00484 PMC4823605

[20] Parsaei M, Sarafraz N, Moaddab SY, Ebrahimzadeh Leylabadlo H. The importance of Faecalibacterium prausnitzii in human health and diseases. New Microbes New Infect. 2021;43:100928. 34430035 10.1016/j.nmni.2021.100928 PMC8365382

[21] Tessler M, Neumann JS, Afshinnekoo E, Pineda M, Hersch R, Velho LFM, et al. Large-scale differences in microbial biodiversity discovery between 16S amplicon and shotgun sequencing. Sci Rep. 2017;7:6589. 28761145 10.1038/s41598-017-06665-3 PMC5537354

[22] de Vries J, Saleem F, Li E, Chan AWY, Naphtali J, Naphtali P, et al. Comparative analysis of metagenomic (amplicon and shotgun) DNA sequencing to characterize microbial communities in household on-site wastewater treatment systems. Water. 2023;15(2):271. 10.3390/w15020271

[23] Bars-Cortina D, Moratalla-Navarro F, García-Serrano A, Mach N, Riobó-Mayo L, Vea-Barbany J, et al. Improving species level-taxonomic assignment from 16S rRNA sequencing technologies. Curr Protocols. 2023;3(11):e930. 10.1002/cpz1.930 37988265

[24] Ilett EE, Jørgensen M, Noguera-Julian M, Daugaard G, Murray DD, Helleberg M, et al. Gut microbiome comparability of fresh-frozen versus stabilized-frozen samples from hospitalized patients using 16S rRNA gene and shotgun metagenomic sequencing. Sci Rep. 2019;9(1):13351. 31527823 10.1038/s41598-019-49956-7 PMC6746779

[25] Salava A, Deptula P, Lyyski A, Laine P, Paulin L, Väkevä L, et al. Skin microbiome in cutaneous T-cell lymphoma by 16S and whole-genome shotgun sequencing. J Invest Dermatol. 2020;140(11):2304–e23087. 32353450 10.1016/j.jid.2020.03.951

[26] Durazzi F, Sala C, Castellani G, Manfreda G, Remondini D, De Cesare A. Comparison between 16S rRNA and shotgun sequencing data for the taxonomic characterization of the gut microbiota. Sci Rep. 2021;11(1):3030. 33542369 10.1038/s41598-021-82726-y PMC7862389

[27] Zuo W, Wang B, Bai X, Luan Y, Fan Y, Michail S, et al. 16S rRNA and metagenomic shotgun sequencing data revealed consistent patterns of gut microbiome signature in pediatric ulcerative colitis. Sci Rep. 2022;12(1):6421. 35440670 10.1038/s41598-022-07995-7 PMC9018687

[28] Hannigan GD, Duhaime MB, Ruffin MT, Koumpouras CC, Schloss PD. Diagnostic potential and interactive dynamics of the colorectal cancer virome. mBio. 2018;9(6). 10.1128/mbio.02248-18 10.1128/mBio.02248-18 PMC6247079 30459201

[29] Nagata N, Nishijima S, Kojima Y, Hisada Y, Imbe K, Miyoshi-Akiyama T, et al. Metagenomic identification of microbial signatures predicting pancreatic cancer from a multinational study. Gastroenterology. 2022;163(1):222–38. 35398347 10.1053/j.gastro.2022.03.054

[30] Castells A, Andreu M, Binefa G, Fité A, Font R, Espinàs JA. Postpolypectomy surveillance in patients with adenomas and serrated lesions: a proposal for risk stratification in the context of organized colorectal cancer-screening programs. Endoscopy. 2015;47(1):86–7. 25532113 10.1055/s-0034-1378100

[31] Rius-Sansalvador B, Bars-Cortina D, Khannous-Lleiffe O, Serrano AG, Guinó E, Saus E et al. Stability of oral and fecal microbiome at room temperature: impact on diversity. bioRxiv. 2023 [cited 2023 Dec 18]:2023.11.28.568988. https://www.biorxiv.org/content/10.1101/2023.11.28.568988v1 10.3389/frmbi.2025.1334775 PMC12993597 41852417

[32] Khannous-Lleiffe O, Willis JR, Saus E, Moreno V, Castellví-Bel S, Gabaldón T, et al. Microbiome profiling from fecal immunochemical test reveals microbial signatures with potential for colorectal cancer screening. Cancers (Basel). 2022;15(1):120. 36612118 10.3390/cancers15010120 PMC9817783

[33] Callahan BJ, McMurdie PJ, Rosen MJ, Han AW, Johnson AJA, Holmes SP. DADA2: high-resolution sample inference from illumina amplicon data. Nat Methods. 2016;13(7):581–3. 27214047 10.1038/nmeth.3869 PMC4927377

[34] Almeida A, Nayfach S, Boland M, Strozzi F, Beracochea M, Shi ZJ, et al. A unified catalog of 204,938 reference genomes from the human gut microbiome. Nat Biotechnol. 2021;39(1):105–14. 32690973 10.1038/s41587-020-0603-3 PMC7801254

[35] Gloor GB, Macklaim JM, Pawlowsky-Glahn V, Egozcue JJ. Microbiome datasets are compositional: and this is not optional. Frontiers in Microbiology. 2017 [cited 2023 Dec 19];8. https://www.frontiersin.org/articles/10.3389/fmicb.2017.02224 10.3389/fmicb.2017.02224 PMC5695134 29187837

[36] Pawlowsky-Glahn V, Egozcue JJ. Compositional data and their analysis: an introduction. Geol Soc Lond Special Publications. 2006;264(1):1–10. 10.1144/GSL.SP.2006.264.01.01

[37] Sim J, Wright CC. The kappa statistic in reliability studies: use, interpretation, and sample size requirements. Phys Ther. 2005;85(3):257–68. 15733050 10.1093/ptj/85.3.257

[38] Bray JR, Curtis JT. An ordination of the upland forest communities of Southern Wisconsin. Ecol Monogr. 1957;27(4):326–49. 10.2307/1942268

[39] Neu AT, Allen EE, Roy K. Defining and quantifying the core microbiome: challenges and prospects. Proceedings of the National Academy of Sciences. 2021;118(51):e2104429118. 10.1073/pnas.2104429118 PMC8713806 34862327

[40] Willis AD, Rarefaction. Alpha diversity, and statistics. Frontiers in Microbiology. 2019 [cited 2022 May 12];10. https://www.frontiersin.org/article/10.3389/fmicb.2019.02407 10.3389/fmicb.2019.02407 PMC6819366 31708888

[41] Hong J, Karaoz U, de Valpine P, Fithian W. To rarefy or not to rarefy: robustness and efficiency trade-offs of rarefying microbiome data. Bioinformatics. 2022;btac127. 10.1093/bioinformatics/btac127 35212706

[42] Tekwa EW, Whalen MA, Martone PT, O’Connor MI. Theory and application of an improved species richness estimator. Philosophical Trans Royal Soc B: Biol Sci. 2023;378(1881):20220187. 10.1098/rstb.2022.0187 PMC10225866 37246376

[43] Pripp AH. Pearsons eller Spearmans korrelasjonskoeffisienter. Tidsskrift for Den norske legeforening. 2018 [cited 2023 Nov 17]; https://tidsskriftet.no/2018/05/medisin-og-tall/pearsons-eller-spearmans-korrelasjonskoeffisienter 10.4045/tidsskr.18.0042 29737766

[44] Ranjan R, Rani A, Metwally A, McGee HS, Perkins DL. Analysis of the microbiome: advantages of whole genome shotgun versus 16S amplicon sequencing. Biochem Biophys Res Commun. 2016;469(4):967–77. 26718401 10.1016/j.bbrc.2015.12.083 PMC4830092

[45] Laudadio I, Fulci V, Palone F, Stronati L, Cucchiara S, Carissimi C. Quantitative assessment of shotgun metagenomics and 16S rDNA amplicon sequencing in the study of human gut microbiome. OMICS. 2018;22(4):248–54. 29652573 10.1089/omi.2018.0013

[46] Fultz R, Ticer T, Ihekweazu FD, Horvath TD, Haidacher SJ, Hoch KM, et al. Unraveling the metabolic requirements of the gut commensal Bacteroides ovatus. Front Microbiol. 2021;12:745469. 34899632 10.3389/fmicb.2021.745469 PMC8656163

[47] Mas-Lloret J, Obón-Santacana M, Ibáñez-Sanz G, Guinó E, Pato ML, Rodriguez-Moranta F, et al. Gut microbiome diversity detected by high-coverage 16S and shotgun sequencing of paired stool and colon sample. Sci Data. 2020;7(1):92. 32179734 10.1038/s41597-020-0427-5 PMC7075950

[48] Morotomi M, Nagai F, Sakon H, Tanaka R. Dialister succinatiphilus sp. nov. and Barnesiella intestinihominis sp. nov., isolated from human faeces. Int J Syst Evol Microbiol. 2008;58(Pt 12):2716–20. 19060046 10.1099/ijs.0.2008/000810-0

[49] Yao S, Zhao Z, Wang W, Liu X. Bifidobacterium longum: protection against inflammatory bowel disease. J Immunol Res. 2021;2021:8030297. 34337079 10.1155/2021/8030297 PMC8324359

[50] Coker OO, Wu WKK, Wong SH, Sung JJY, Yu J. Altered gut archaea composition and interaction with bacteria are associated with colorectal cancer. Gastroenterology. 2020;159(4):1459–e14705. 32569776 10.1053/j.gastro.2020.06.042

[51] Welham Z, Li J, Engel AF, Molloy MP. Mucosal microbiome in patients with early bowel polyps: inferences from short-read and long-read 16S rRNA sequencing. Cancers (Basel). 2023;15(20):5045. 37894412 10.3390/cancers15205045 PMC10605900

[52] Yi S, Zhang C, Yin P, Yu L, Tian F, Chen W, et al. Compositional and functional features of the intestinal lactobacilli associated with different long-term diet types. Food Funct. 2023;14(14):6570–81. 37382555 10.1039/D3FO02182C

[53] McDonald D, Jiang Y, Balaban M, Cantrell K, Zhu Q, Gonzalez A et al. Greengenes2 unifies microbial data in a single reference tree. Nat Biotechnol. 2023:1–4. 10.1038/s41587-023-01845-1 PMC10818020 37500913

