# Supplementary figures and images for: Comparison between 16S rRNA and shotgun sequencing in colorectal cancer, advanced colorectal lesions, and healthy human gut microbiota
Source: BMC Genomics. 2024 Jul 29;25:730. doi: 10.1186/s12864-024-10621-7 (PMC11285316; doi:10.1186/s12864-024-10621-7)

**Additional Figure S1.** Rarefaction curves for shotgun and 16S. In red the minimum depth.

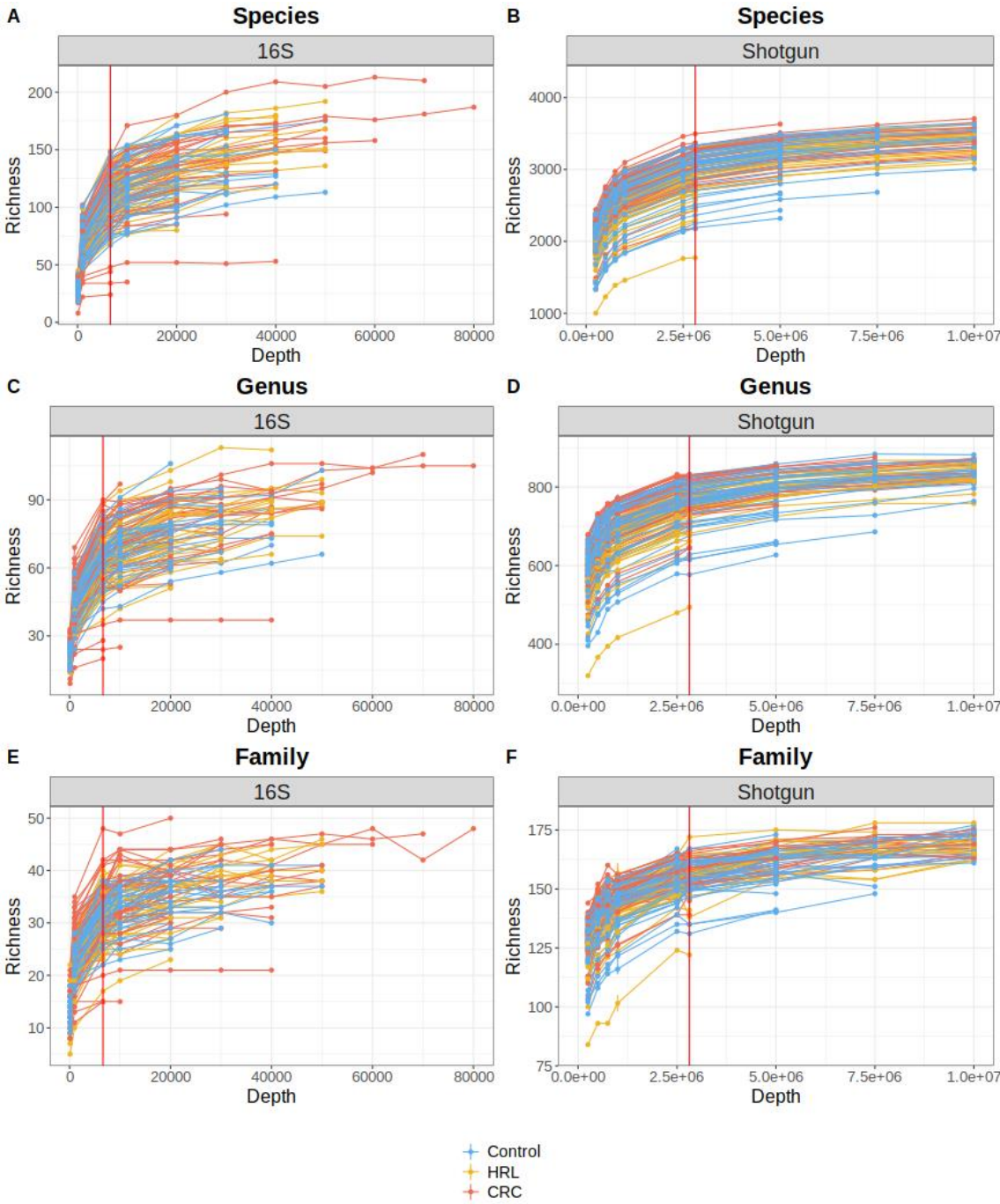

Supplement: Supplementary file 1 — Supplementary Material 1 [file 12864_2024_10621_MOESM1_ESM.pdf]

**Additional Figure 2.** Heatmaps of the top 50 most abundant taxa (on average) shared by shotgun and 16S

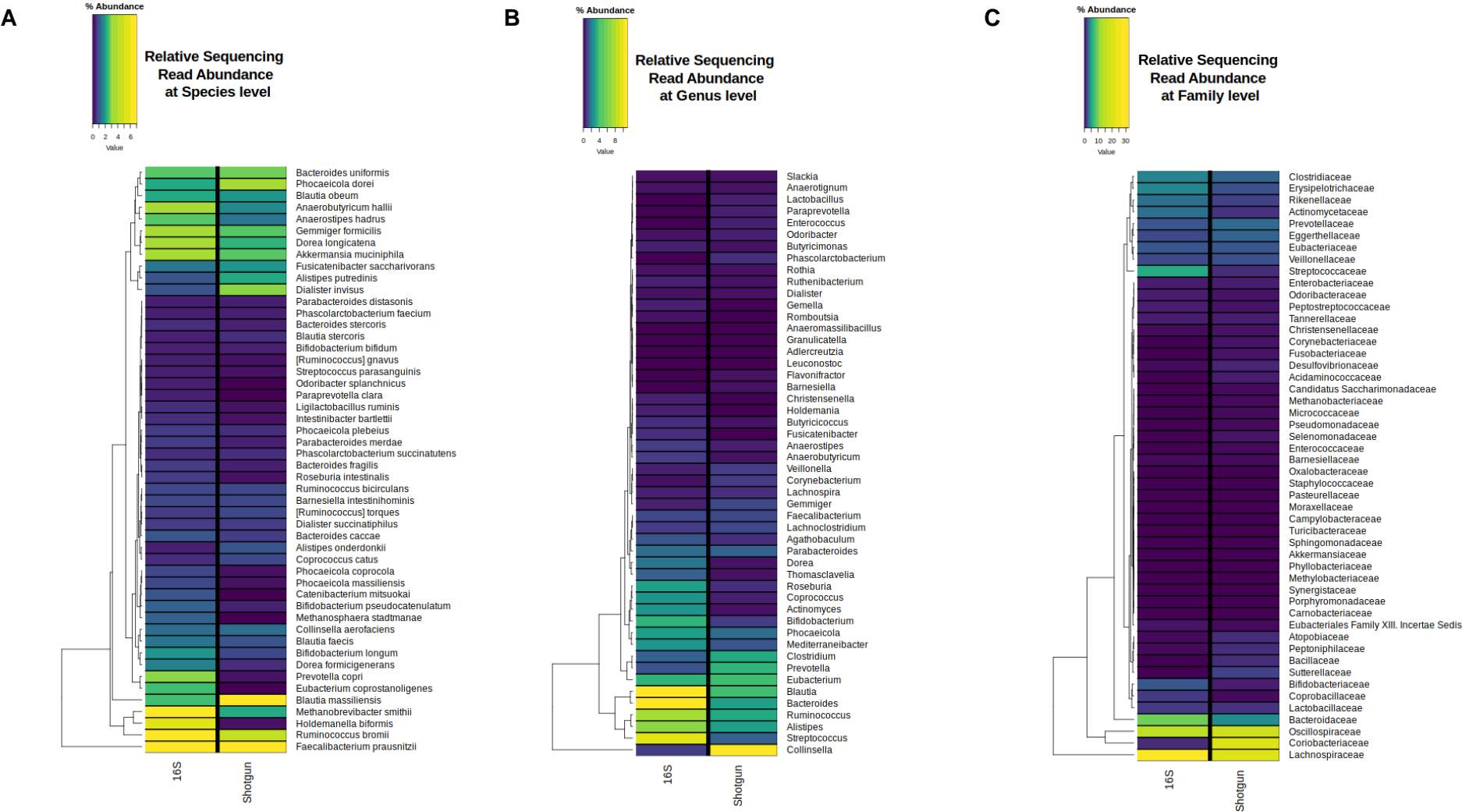

Supplement: Supplementary file 2 — Supplementary Material 2 [file 12864_2024_10621_MOESM2_ESM.pdf]
