# Supplementary material for: Comparison between 16S rRNA and shotgun sequencing in colorectal cancer, advanced colorectal lesions, and healthy human gut microbiota
Source: BMC Genomics. 2024 Jul 29;25:730. doi: 10.1186/s12864-024-10621-7 (PMC11285316; doi:10.1186/s12864-024-10621-7)
Supplement: Supplementary file 3 — Supplementary Material 3 [file 12864_2024_10621_MOESM3_ESM.pdf]

**Additional Figure S3.** Bar plots of the top 15 most common genera and families in 16S and shotgun abundance data. Each row represents a different sample with the same order in both plots

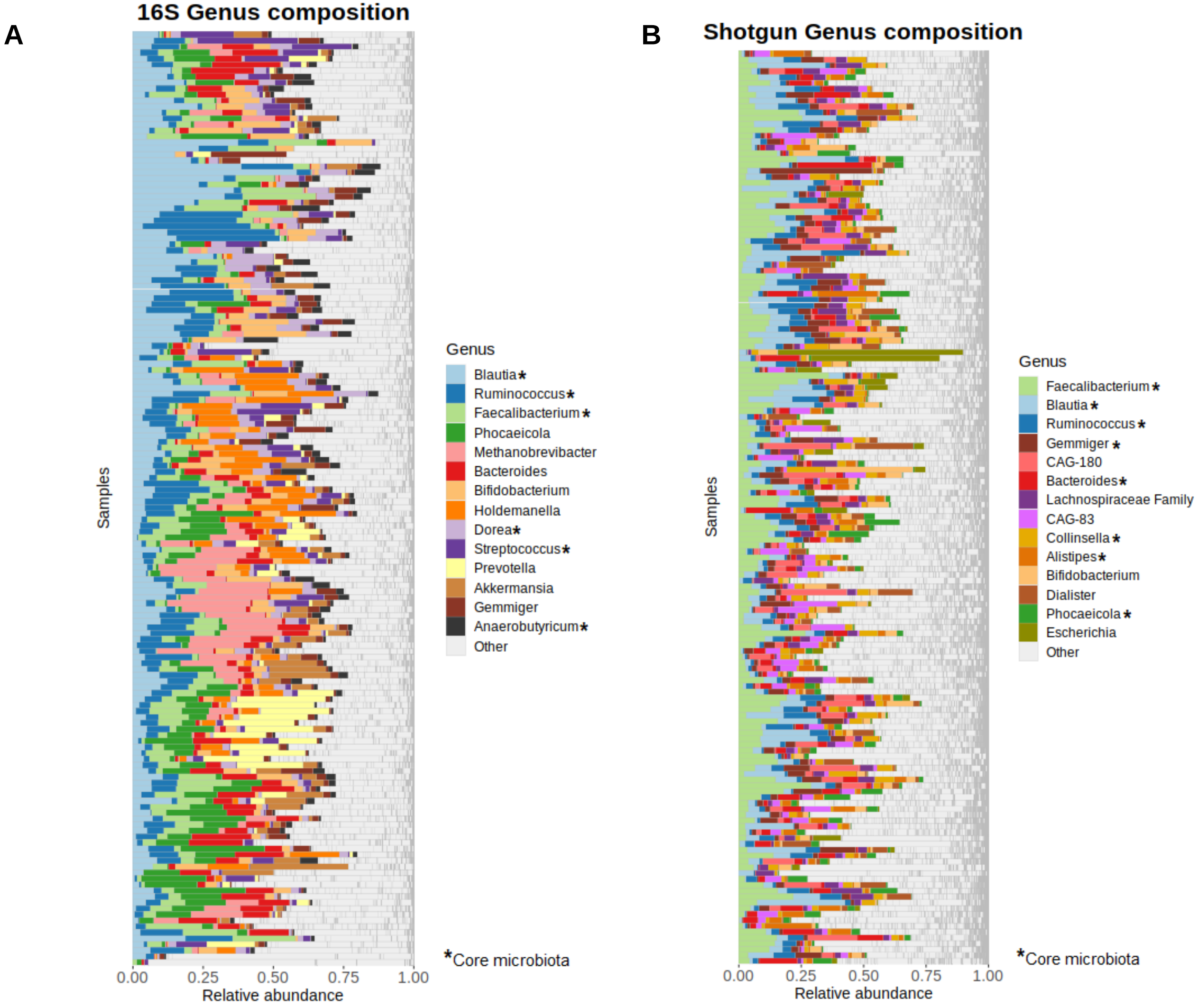

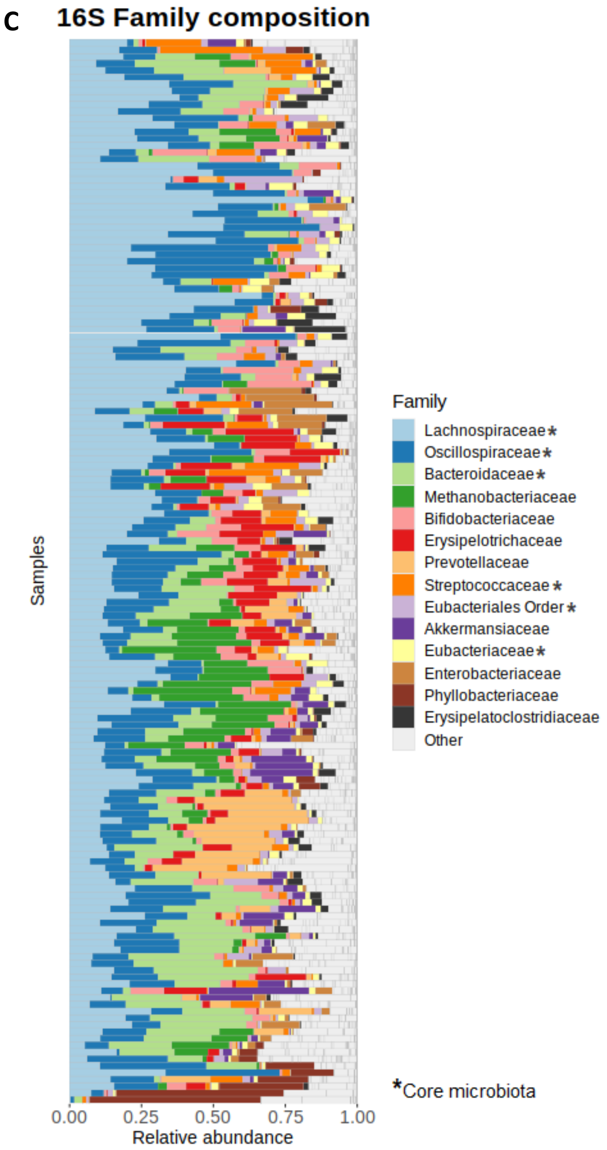
C
16S Family composition
Samples
Relative abundance
0.00
0.25
0.50
0.75
1.00
Family
Lachnospiraceae *
Oscillospiraceae *
Bacteroidaceae *
Methanobacteriaceae
Bifidobacteriaceae
Erysipelotrichaceae
Prevotellaceae
Streptococcaceae *
Eubacteriales Order *
Akkermansiaceae
Eubacteriaceae *
Enterobacteriaceae
Phyllobacteriaceae
Erysipelatoclostridiaceae
Other
*Core microbiota

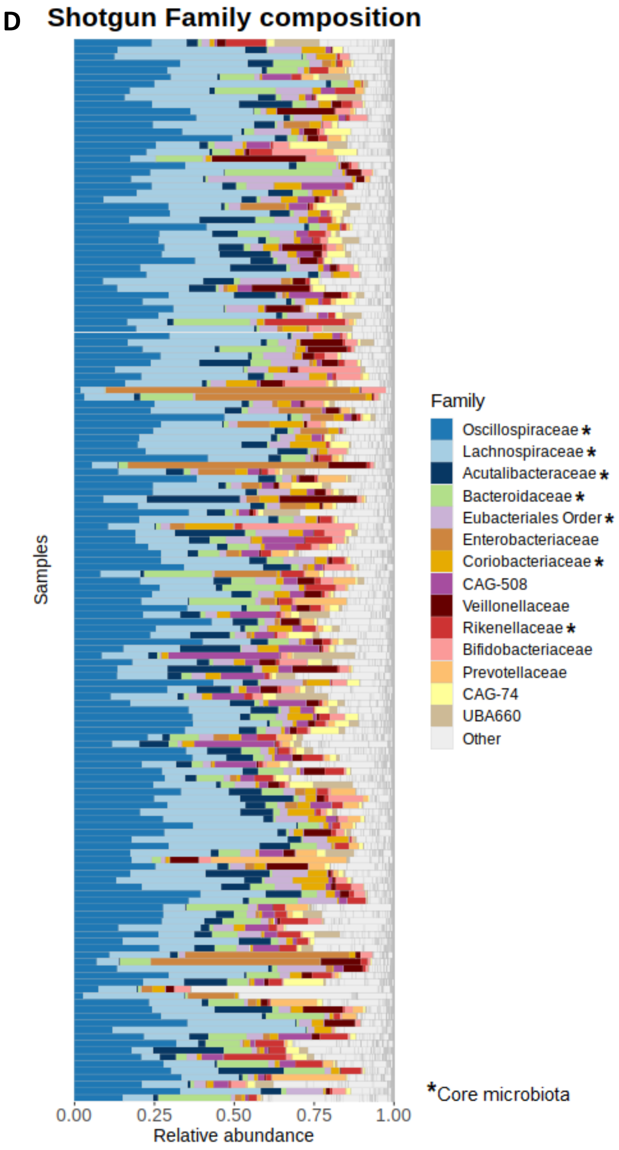
D
Shotgun Family composition
Samples
Relative abundance
0.00
0.25
0.50
0.75
1.00
Family
Oscillospiraceae *
Lachnospiraceae *
Acutalibacteraceae *
Bacteroidaceae *
Eubacteriales Order *
Enterobacteriaceae
Coriobacteriaceae *
CAG-508
Veillonellaceae
Rikenellaceae *
Bifidobacteriaceae
Prevotellaceae
CAG-74
UBA660
Other
*Core microbiota
