# Supplementary material for: Comparison between 16S rRNA and shotgun sequencing in colorectal cancer, advanced colorectal lesions, and healthy human gut microbiota
Source: BMC Genomics. 2024 Jul 29;25:730. doi: 10.1186/s12864-024-10621-7 (PMC11285316; doi:10.1186/s12864-024-10621-7)

Additional Figure 6 Importance of the Family models (top-10). Species common to 16S and shotgun signatures are highlighted in blue.

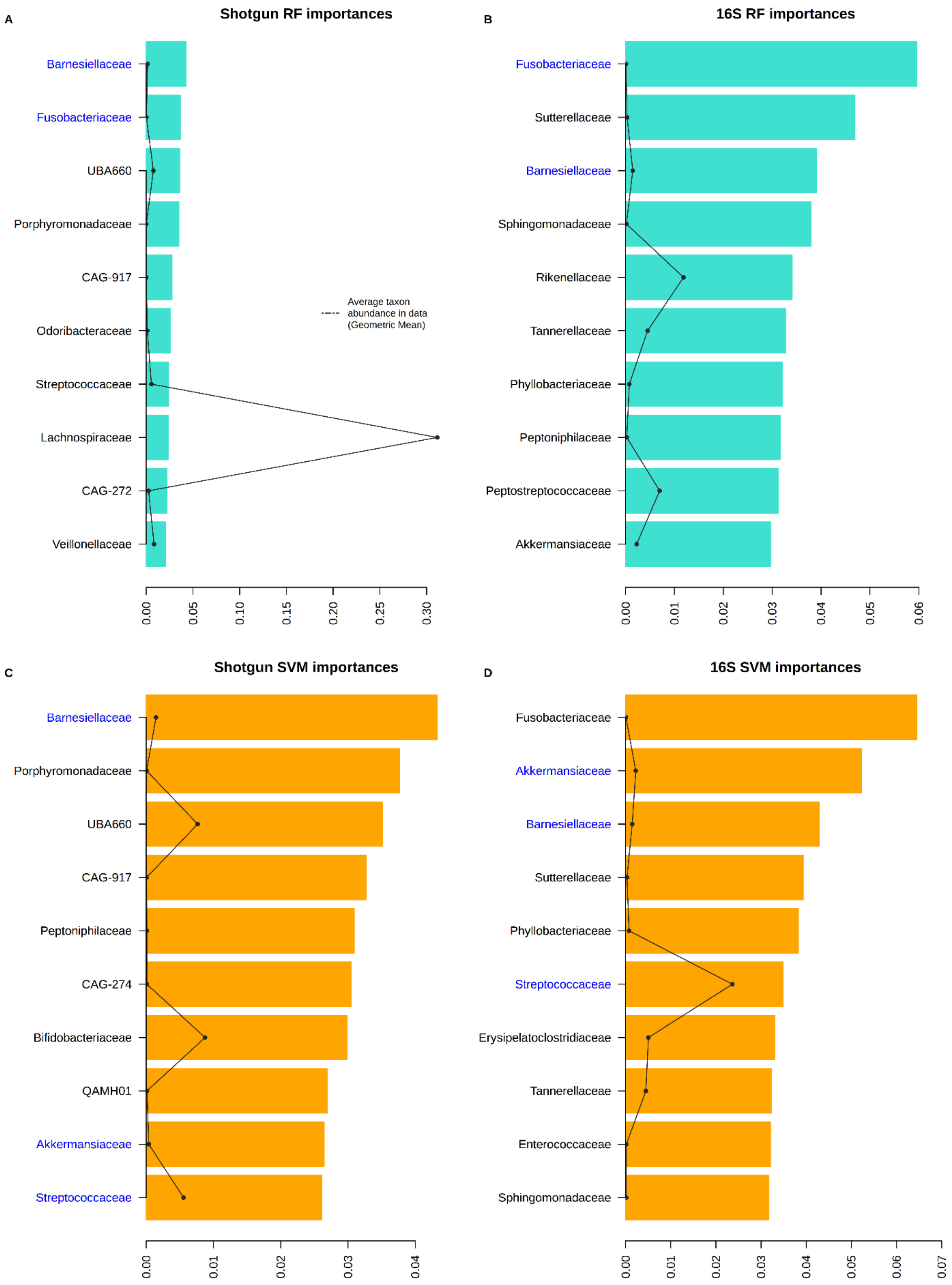

Supplement: Supplementary file 6 — Supplementary Material 6 [file 12864_2024_10621_MOESM6_ESM.pdf]
