# Supplementary material for: Comparison between 16S rRNA and shotgun sequencing in colorectal cancer, advanced colorectal lesions, and healthy human gut microbiota
Source: BMC Genomics. 2024 Jul 29;25:730. doi: 10.1186/s12864-024-10621-7 (PMC11285316; doi:10.1186/s12864-024-10621-7)
Supplement: Supplementary file 7 — Supplementary Material 7 [file 12864_2024_10621_MOESM7_ESM.pdf]

Additional Figure 7 Importance of SVM binary models at the Species level (top-50). The agreement between 16S and shotgun in the Control vs HRL signatures is bounded between 16%-26%, 12%-26% in HRL vs CRC, and 22%-28% in Control vs CRC. Species common to 16S and shotgun signatures are highlighted in blue.

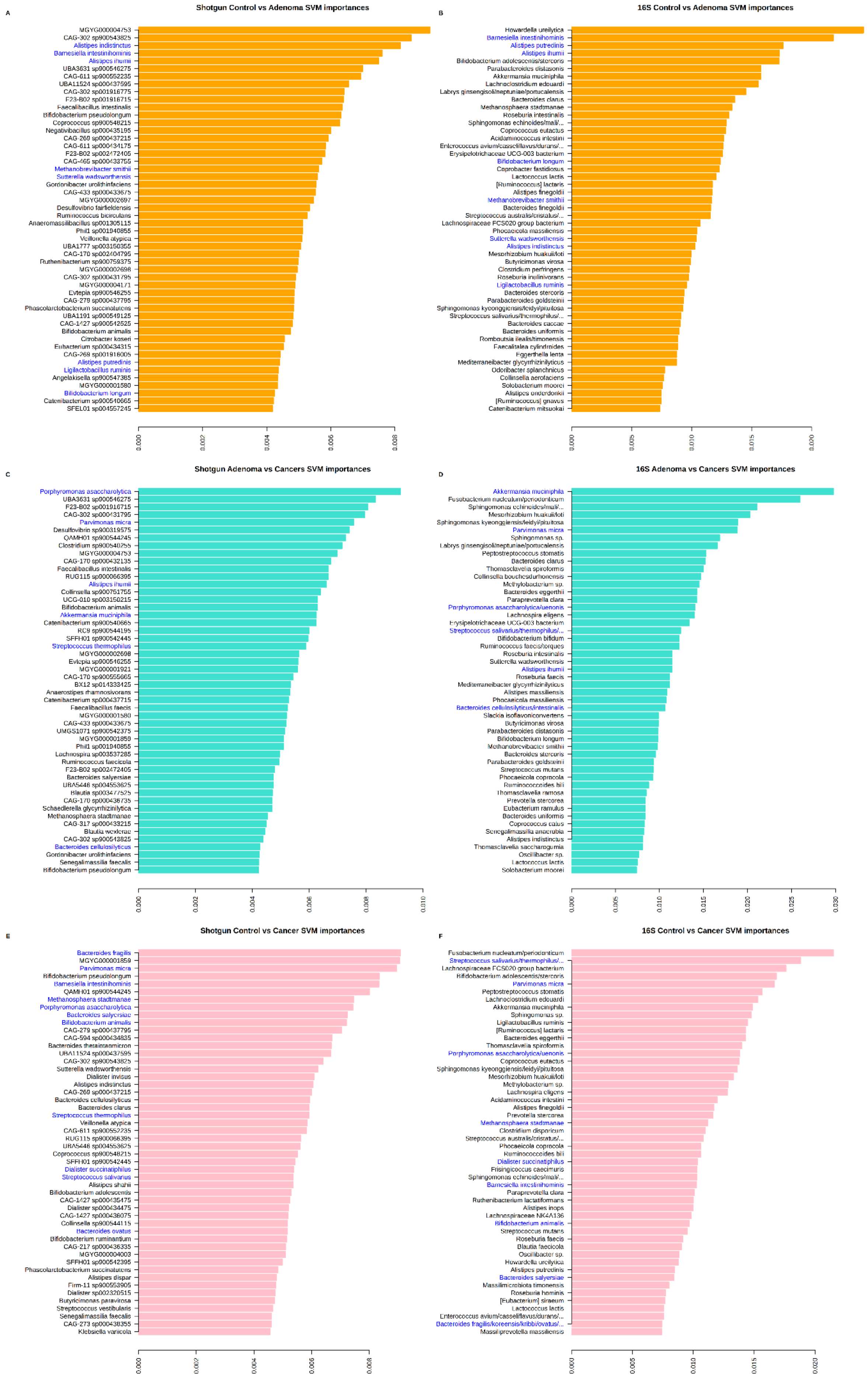
A
Shotgun Control vs Adenoma SVM importances
MGYG000004753
CAG-302 sp900543825
Alistipes indistinctus
Barnesiella intestinihominis
Alistipes ihumii
UBA3631 sp900546275
CAG-611 sp900552235
UBA11524 sp000437595
CAG-302 sp001916775
F23-B02 sp001916715
Faecalibacillus intestinalis
Bifidobacterium pseudolongum
Coprococcus sp900548215
Negativibacillus sp000435195
CAG-269 sp000437215
CAG-611 sp000434175
F23-B02 sp002472405
CAG-465 sp000433755
Methanobrevibacter smithii
Sutterella wadsworthensis
Gordonibacter urolithinfaciens
CAG-433 sp000433675
MGYG000002697
Desulfovibrio fairfieldensis
Ruminococcus bicirculans
Anaeromassilibacillus sp001305115
Phil1 sp001940855
Veillonella atypica
UBA1777 sp003150355
CAG-170 sp002404795
Ruthenibacterium sp900759375
MGYG000002698
CAG-302 sp000431795
MGYG000004171
Evtepia sp900546255
CAG-279 sp000437795
Phascolarctobacterium succinatutens
UBA1191 sp900549125
CAG-1427 sp900542525
Bifidobacterium animalis
Citrobacter koseri
Eubacterium sp000434315
CAG-269 sp001916005
Alistipes putredinis
Ligilactobacillus ruminis
Angelakisella sp900547385
MGYG000001580
Bifidobacterium longum
Catenibacterium sp900540665
SFEL01 sp004557245
0.000
0.002
0.004
0.006
0.008
B
16S Control vs Adenoma SVM importances
Howardella ureilytica
Barnesiella intestinihominis
Alistipes putredinis
Alistipes ihumii
Bifidobacterium adolescentis/stercoris
Parabacteroides distasonis
Akkermansia muciniphila
Lachnoclostridium edouardi
Labrys ginsengisoli/neptuniae/portucalensis
Bacteroides clarus
Methanosphaera stadtmanae
Roseburia intestinalis
Sphingomonas echinoides/mali/...
Coprococcus eutactus
Acidaminococcus intestini
Enterococcus avium/casseliflavus/durans/...
Erysipelotrichaceae UCG-003 bacterium
Bifidobacterium longum
Coprobacter fastidiosus
Lactococcus lactis
[Ruminococcus] lactaris
Alistipes finegoldii
Methanobrevibacter smithii
Bacteroides finegoldii
Streptococcus australis/cristatus/...
Lachnospiraceae FCS020 group bacterium
Phocaeicola massiliensis
Sutterella wadsworthensis
Alistipes indistinctus
Mesorhizobium huakuii/loti
Butyricimonas virosa
Clostridium perfringens
Roseburia inulinivorans
Ligilactobacillus ruminis
Bacteroides stercoris
Parabacteroides goldsteinii
Sphingomonas kyeongjiensis/leidyi/pituitosa
Streptococcus salivarius/thermophilus/...
Bacteroides caccae
Bacteroides uniformis
Romboutsia ilealis/timonensis
Faecalitalea cylindroides
Eggerthella lenta
Mediterraneibacter glycyrrhizinilyticus
Odoribacter splanchnicus
Collinsella aerofaciens
Solobacterium moorei
Alistipes onderdonkii
[Ruminococcus] gnavus
Catenibacterium mitsuokai
0.000
0.005
0.010
0.015
0.020
C
Shotgun Adenoma vs Cancers SVM importances
Porphyromonas asaccharolytica
UBA3631 sp900546275
F23-B02 sp001916715
CAG-302 sp000431795
Parvimonas micra
Desulfovibrio sp900319575
QAMH01 sp900544245
Clostridium sp900540255
MGYG000004753
CAG-170 sp000432135
Faecalibacillus intestinalis
RUG115 sp900066395
Alistipes ihumii
Collinsella sp900751755
UCG-010 sp003150215
Bifidobacterium animalis
Akkermansia muciniphila
Catenibacterium sp900540665
RC9 sp900544195
SFFH01 sp900542445
Streptococcus thermophilus
MGYG000002698
Evtepia sp900546255
MGYG000001921
CAG-170 sp900555665
BX12 sp014333425
Anaerostipes rhamnosivorans
Catenibacterium sp000437715
Faecalibacillus faecis
MGYG000001580
CAG-433 sp000433675
UMGS1071 sp900542375
MGYG000001859
Phil1 sp001940855
Lachnospira sp003537285
Ruminococcus faecicola
F23-B02 sp002472405
Bacteroides salyersiae
UBA5446 sp004553625
Blautia sp003477525
CAG-170 sp000436735
Schaedlerella glycyrrhizinilytica
Methanosphaera stadtmanae
CAG-317 sp000433215
Blautia wexlerae
CAG-302 sp900543825
Bacteroides cellulosilyticus
Gordonibacter urolithinfaciens
Senegalimassilia faecalis
Bifidobacterium pseudolongum
0.000
0.002
0.004
0.006
0.008
0.010
D
16S Adenoma vs Cancers SVM importances
Akkermansia muciniphila
Fusobacterium nucleatum/periodonticum
Sphingomonas echinoides/mali/...
Mesorhizobium huakuii/loti
Sphingomonas kyeongjiensis/leidyi/pituitosa
Parvimonas micra
Sphingomonas sp.
Labrys ginsengisoli/neptuniae/portucalensis
Peptostreptococcus stomatis
Bacteroides clarus
Thomasclavelia spiroformis
Collinsella bouchesdurhonensis
Methylobacterium sp.
Bacteroides eggerthii
Paraprevotella clara
Porphyromonas asaccharolytica/uenonis
Lachnospira eligens
Erysipelotrichaceae UCG-003 bacterium
Streptococcus salivarius/thermophilus/...
Bifidobacterium bifidum
Ruminococcus faecis/torques
Roseburia intestinalis
Sutterella wadsworthensis
Alistipes ihumii
Roseburia faecis
Mediterraneibacter glycyrrhizinilyticus
Alistipes massiliensis
Phocaeicola massiliensis
Bacteroides cellulosilyticus/intestinalis
Slackia isoflavoniconvertens
Butyricimonas virosa
Parabacteroides distasonis
Bifidobacterium longum
Methanobrevibacter smithii
Bacteroides stercoris
Parabacteroides goldsteinii
Streptococcus mutans
Phocaeicola coprocola
Ruminococcoides bili
Thomasclavelia ramosa
Prevotella stercorea
Eubacterium ramulus
Bacteroides uniformis
Coprococcus catus
Senegalimassilia anaerobia
Alistipes indistinctus
Thomasclavelia saccharogumia
Oscillibacter sp.
Lactococcus lactis
Solobacterium moorei
0.000
0.005
0.010
0.015
0.020
0.025
0.030
E
Shotgun Control vs Cancer SVM importances
Bacteroides fragilis
MGYG000001859
Parvimonas micra
Bifidobacterium pseudolongum
Barnesiella intestinihominis
QAMH01 sp900544245
Methanosphaera stadtmanae
Porphyromonas asaccharolytica
Bacteroides salyersiae
Bifidobacterium animalis
CAG-279 sp000437795
CAG-594 sp000434835
Bacteroides thetaiotaomicron
UBA11524 sp000437595
CAG-302 sp900543825
Sutterella wadsworthensis
Dialister invisus
Alistipes indistinctus
CAG-269 sp000437215
Bacteroides cellulosilyticus
Bacteroides clarus
Streptococcus thermophilus
Veillonella atypica
CAG-611 sp900552235
RUG115 sp900066395
UBA5446 sp004553625
Coprococcus sp900548215
SFFH01 sp900542445
Dialister succinatiphilus
Streptococcus salivarius
Alistipes shahii
Bifidobacterium adolescentis
CAG-1427 sp000435475
Dialister sp000434475
CAG-1427 sp000436075
Collinsella sp900544115
Bacteroides ovatus
Bifidobacterium ruminantium
CAG-217 sp000436335
MGYG000004003
SFFH01 sp900542395
Phascolarctobacterium succinatutens
Alistipes dispar
Firm-11 sp900553905
Dialister sp002320515
Butyricimonas paravirosa
Streptococcus vestibularis
Senegalimassilia faecalis
CAG-273 sp000438355
Klebsiella variicola
0.000
0.002
0.004
0.006
0.008
F
16S Control vs Cancer SVM importances
Fusobacterium nucleatum/periodonticum
Streptococcus salivarius/thermophilus/...
Lachnospiraceae FCS020 group bacterium
Bifidobacterium adolescentis/stercoris
Parvimonas micra
Peptostreptococcus stomatis
Lachnoclostridium edouardi
Akkermansia muciniphila
Sphingomonas sp.
Ligilactobacillus ruminis
[Ruminococcus] lactaris
Bacteroides eggerthii
Thomasclavelia spiroformis
Porphyromonas asaccharolytica/uenonis
Coprococcus eutactus
Sphingomonas kyeongjiensis/leidyi/pituitosa
Mesorhizobium huakuii/loti
Methylobacterium sp.
Lachnospira eligens
Acidaminococcus intestini
Alistipes finegoldii
Prevotella stercorea
Methanosphaera stadtmanae
Clostridium disporicum
Streptococcus australis/cristatus/...
Phocaeicola coprocola
Ruminococcoides bili
Dialister succinatiphilus
Frisingicoccus caecimuris
Sphingomonas echinoides/mali/...
Barnesiella intestinihominis
Paraprevotella clara
Ruthenibacterium lactatiformans
Alistipes inops
Lachnospiraceae NK4A136
Bifidobacterium animalis
Streptococcus mutans
Roseburia faecis
Blautia faecicola
Oscillibacter sp.
Howardella ureilytica
Alistipes putredinis
Bacteroides salyersiae
Massilimicrobiota timonensis
Roseburia hominis
[Eubacterium] siraeum
Lactococcus lactis
Enterococcus avium/casseliflavus/durans/...
Bacteroides fragilis/koreensis/kribbi/ovatus/...
Massiliprevotella massiliensis
0.000
0.005
0.010
0.015
0.020

Note: Complete names of the 16S taxa that have been truncated in the plot:

- *Enterococcus avium/casseliflavus/durans/faecium/gallinarum/hermanniensis/hirae/hiraes/phoeniculicola/raffinosus/saccharolyticus/thailandicus*
- *Streptococcus salivarius/thermophilus/vestibularis*
- *Streptococcus australis/cristatus/gordonii/infantis/mitis/oralis/parasanguinis/pneumoniae/pseudopneumoniae/sanguinis*
- *Bacteroides fragilis/koreensis/kribbi/ovatus/xylanisolvens*
- *Sphingomonas echinoides/mali/oligophenolica/sanxanigenens*
