# Supplementary material for: Comparison between 16S rRNA and shotgun sequencing in colorectal cancer, advanced colorectal lesions, and healthy human gut microbiota
Source: BMC Genomics. 2024 Jul 29;25:730. doi: 10.1186/s12864-024-10621-7 (PMC11285316; doi:10.1186/s12864-024-10621-7)
Supplement: Supplementary file 8 — Supplementary Material 8 [file 12864_2024_10621_MOESM8_ESM.pdf]

Additional Table 1 Wilcoxon Rank Sum Tests' p-values comparing the alpha diversity distribution between 16S and shotgun, stratified by diagnostic status at the species, genus and family taxonomy levels.

Wilcoxon p-value	Species		Genus		Family	
	**Shannon**	**Chao 1**	**Shannon**	**Chao 1**	**Shannon**	**Chao 1**
Negative	< 2.2e-16	< 2.2e-16	4.1e-15	< 2.2e-16	0.020	< 2.2e-16
HRL	5.6e-15	< 2.2e-16	1.1e-11	< 2.2e-16	0.020	< 2.2e-16
CRC	< 2.2e-16	< 2.2e-16	3.3e-15	< 2.2e-16	0.001	< 2.2e-16
