# Supplementary material for: Comparison between 16S rRNA and shotgun sequencing in colorectal cancer, advanced colorectal lesions, and healthy human gut microbiota
Source: BMC Genomics. 2024 Jul 29;25:730. doi: 10.1186/s12864-024-10621-7 (PMC11285316; doi:10.1186/s12864-024-10621-7)
Supplement: Supplementary file 9 — Supplementary Material 9 [file 12864_2024_10621_MOESM9_ESM.pdf]

Additional Table 2 Kruskal-Wallis p-value for the distribution of zeros (see **Figure 6)** in controls, HRL and CRC patients, before and after filtering.

**Kruskal Wallis**	**Species**	**Genus**	**Family**
shotgun	0.044	0.029	0.15
16S	0.77	0.88	0.61
shotgun (Filtered)	0.014	0.006	0.25
16S (Filtered)	0.67	0.91	0.36
