# Supplementary material for: Comparison between 16S rRNA and shotgun sequencing in colorectal cancer, advanced colorectal lesions, and healthy human gut microbiota
Source: BMC Genomics. 2024 Jul 29;25:730. doi: 10.1186/s12864-024-10621-7 (PMC11285316; doi:10.1186/s12864-024-10621-7)
Supplement: Supplementary file 10 — Supplementary Material 10 [file 12864_2024_10621_MOESM10_ESM.pdf]

Additional Table 3 PERMANOVA results and its post-hoc analysis. 9999 permutations. All the p-values are Benjamini-Hochberg adjusted p-values. C: control, HRL, CRC.

	**16S**		**Shotgun**	
	PERMANOVA	Post-hoc	PERMANOVA	Post-hoc
**Family**	F=4.4,<br>p-value: 1e-4	C vs HRL: 1.5e-4<br>C vs CRC: 3.1e-3<br>HRL vs CRC: 1.5e-4	F=3.3,<br>p-value: 1e-4	C vs HRL: 2.5e-3<br>C vs CRC: 4.5e-4<br>HRL vs CRC: 4.5e-4
**Genus**	F=2.8,<br>p-value: 1e-4	C vs HRL: 1.5e-4<br>C vs CRC: 3.4e-3<br>HRL vs CRC: 1.5e-4	F=2.7,<br>p-value: 2e-4	C vs HRL: 6e-4<br>C vs CRC: 0.01<br>HRL vs CRC: 4e-4
**Species**	F=4.4,<br>p-value: 1e-4	C vs HRL: 1.5e-4<br>C vs CRC: 1.4e-3<br>HRL vs CRC: 1.5e-4	F=2.2,<br>p-value: 1e-4	C vs HRL: 1.4e-3<br>C vs CRC: 1.8e-3<br>HRL vs CRC: 1.4e-3
