# Supplementary material for: Comparison between 16S rRNA and shotgun sequencing in colorectal cancer, advanced colorectal lesions, and healthy human gut microbiota
Source: BMC Genomics. 2024 Jul 29;25:730. doi: 10.1186/s12864-024-10621-7 (PMC11285316; doi:10.1186/s12864-024-10621-7)
Supplement: Supplementary file 11 — Supplementary Material 11 [file 12864_2024_10621_MOESM11_ESM.pdf]

Additional Table 4 Training and test agreement for the SVM binary models: proportion of agreement, Cohen's kappa and Pearson *r* between the 16S and shotgun models output. Statistically significant Cohen's kappa and Pearson *r* are highlighted in **bold.**

Models		Agreement		Kappa		Pearson *r*	
		**Training**	**Test**	**Training**	**Test**	**Training**	**Test**
Species	Control vs HRL	0.71	0.60	**0.43 (0.28, 0.59)**	0.23 (-0.09, 0.55)	0.50 (0.36, 0.62)	**0.40 (0.05, 0.67)**
	Control vs CRC	0.85	0.77	**0.69 (0.56, 0.82)**	**0.47 (0.14, 0.80)**	0.79 (0.72, 0.85)	**0.46 (0.12, 0.70)**
	HRL vs CRC	0.72	0.67	**0.38 (0.21, 0.55)**	0.29 (-0.06, 0.63)	0.62 (0.50, 0.72)	0.19 (-0.18, 0.52)
Genus	Control vs HRL	0.66	0.60	**0.34 (0.19, 0.49)**	0.23 (-0.09, 0.54)	0.46 (0.31, 0.59)	**0.44 (0.09, 0.69)**
	Control vs CRC	0.75	0.77	**0.45 (0.29, 0.61)**	0.31 (-0.08, 0.71)	0.714 (0.62, 0.79)	**0.38 (0.03, 0.65)**
	HRL vs CRC	0.71	0.73	**0.34 (0.16, 0.51)**	**0.38 (0.04, 0.72)**	0.60 (0.47, 0.70)	0.23 (-0.14,0.55)
Family	Control vs HRL	0.67	0.67	**0.34 (0.18, 0.50)**	**0.34 (0.003, 0.67)**	0.38 (0.22, 0.52)	**0.44 (0.09, 0.69)**
	Control vs CRC	0.75	0.77	**0.38 (0.20, 0.55)**	0.22 (-0.19, 0.64)	0.69 (0.59, 0.78)	0.25 (-0.12,0.56)
	HRL vs CRC	0.67	0.70	**0.24 (0.06, 0.41)**	0.31 (-0.003, 0.62)	0.52 (0.38, 0.64)	0.13 (-0.24,0.47)
