# Supplementary material for: Comparison between 16S rRNA and shotgun sequencing in colorectal cancer, advanced colorectal lesions, and healthy human gut microbiota
Source: BMC Genomics. 2024 Jul 29;25:730. doi: 10.1186/s12864-024-10621-7 (PMC11285316; doi:10.1186/s12864-024-10621-7)
Supplement: Supplementary file 12 — Supplementary Material 12 [file 12864_2024_10621_MOESM12_ESM.pdf]

Additional Table 5 Agreement in Support Vectors between models (only Cohen's kappa is shown). p-values < 0.05 are in **bold.**

Models		SV vs No SV	p-value	SV(alpha<C) vs SV(alpha=C) vs No SV	p-value
	Control vs HRL	0.05 (-0.20, 0.30)	0.67	0.034 (-0.14, 0.21)	0.71
Species	Control vs CRC	0.22 (-0.11, 0.55)	0.19	**0.31 (0.14, 0.49)**	**5e-4**
	HRL vs CRC	0.077 (-0.170, 0.32)	0.54	**0.18 (0.02, 0.35)**	**0.028**
	Control vs HRL	0.001 (-0.203, 0.21)	0.99	-0.02 (-0.16, 0.12)	0.79
Genus	Control vs CRC	**0.50 (0.203, 0.80)**	**0.001**	**0.42 (0.23, 0.61)**	**1e-5**
	HRL vs CRC	0.15 (-0.10, 0.39)	0.25	**0.22 (0.059, 0.39)**	**7.7e-3**
	Control vs HRL	-0.11 (-0.17, -0.04)	0.28	-0.047 (-0.19, 0.09)	0.71
Family	Control vs CRC	0.20 (-0.063, 0.46)	0.14	**0.25 (0.079, 0.41)**	**3.8e-3**
	HRL vs CRC	**0.24 (0.014, 0.47)**	**0.037**	**0.28 (0.12, 0.44)**	**6.5e-3**
